# A New Strategy to Identify ceRNA-Based CCDC144NL-AS1/SERPINE1 Regulatory Axis as a Novel Prognostic Biomarker for Stomach Adenocarcinoma *via* High Throughput Transcriptome Data Mining and Computational Verification

**DOI:** 10.3389/fonc.2021.802727

**Published:** 2022-01-27

**Authors:** Zhihong Huang, Xinkui Liu, Chao Wu, Shan Lu, Stalin Antony, Wei Zhou, Jingyuan Zhang, Zhishan Wu, Yingying Tan, Xiaotian Fan, Leiming You, Zhiwei Jing, Jiarui Wu

**Affiliations:** ^1^ Department of Clinical Pharmacology of Traditional Chinese Medicine, School of Chinese Materia Medica, Beijing University of Chinese Medicine, Beijing, China; ^2^ Institute of Fundamental and Frontier Sciences, University of Electronic Science and Technology of China, Chengdu, China; ^3^ Pharmacy Department, China-Japan Friendship Hospital, Beijing, China; ^4^ School of Life Science, Beijing University of Chinese Medicine, Beijing, China; ^5^ Institute of Clinical Basic Medicine of Traditional Chinese Medicine, China Academy of Chinese Medicine Science, Beijing, China

**Keywords:** stomach adenocarcinoma, competitive endogenous RNA, CCDC144NL-AS1/SERPINE1 axis, prognostic biomarker, high throughput transcriptome data mining and computational verification

## Abstract

Stomach adenocarcinoma (STAD) is one of the most malignant cancers that endanger human health. There is growing evidence that competitive endogenous RNA (ceRNA) regulatory networks play an important role in various human tumors. However, the complexity and behavioral characteristics of the ceRNA network in STAD are still unclear. In this study, we constructed a ceRNA regulatory network to identify the potential prognostic biomarkers associated with STAD. The expression profile of lncRNA, miRNA, and mRNA was downloaded from The Cancer Genome Atlas (TCGA). After performing bioinformatics analysis, the CCDC144NL-AS1/hsa-miR-145-5p/SERPINE1 ceRNA network associated to STAD prognosis of STAD was obtained. The CCDC144NL-AS1/SERPINE1 axis in the ceRNA network was identified by correlation analysis and considered as a clinical prognosis model by Cox regression analysis. In addition, methylation analysis indicated that the abnormal upregulation of CCDC144NL-AS1/SERPINE1 axis might be related to the aberrant methylation of some sites, and immune infiltration analysis suggested that CCDC144NL-AS1/SERPINE1 axis probably influences the alteration of tumor immune microenvironment and the occurrence and development of STAD. In particular, the CCDC144NL-AS1/SERPINE1 axis based on the ceRNA network constructed in the present study might be an important novel factor correlating with the diagnosis and prognosis of STAD.

## Introduction

Gastric carcinoma (GC), particularly stomach adenocarcinoma (STAD), continues to be of major significant cancer worldwide. With more than 1 million new cases and an estimated 769,000 deaths in 2020, it ranks fifth in incidence and fourth in mortality worldwide (1). Although the curative rate of early-stage STAD is high, the median survival time of advanced stage STAD is only 9-10 months and the 5-year survival rate of STAD is less than 30% (2). STAD is diagnosed by endoscopic examination and staged by CT, endoscopic ultrasound, PET, and laparoscopy. The primary treatment for early-stage STAD is endoscopic resection. Non-early operable STAD is treated with surgery, including D2 lymphadenectomy (3). Risk factors for gastric cancer include many nonmodifiable variables, such as age, gender, race/ethnicity, and other controllable risk factors such as *Helicobacter pylori* infection, smoking, high nitrate, and high nitrite diet (4, 5). At present, the incidence and mortality of STAD continue to increase in many countries, and it is considered to be cancer with a poor prognosis and low survival rate. Therefore, it is necessary to explore useful prognostic biomarkers and/or therapeutic targets for STAD to improve our deficiencies in the diagnosis, prevention, and treatment of the disease.

Long non-coding RNA (lncRNA), a subtype of noncoding RNA (ncRNA), is more than 200 nt in length. MicroRNA (miRNA) is a small single-stranded ncRNA about 22 nt in length. In the past, lncRNA and miRNA were considered incapable of encoding proteins, but recent studies have shown that they are involved in many biological processes (6, 7). Several studies have shown that lncRNA are usually dysregulated in many cancers and are related to cancer recurrence, metastasis, and poor prognosis (8–10). MiRNA can inhibit gene degradation or translation by binding to the 3’ untranslated region (UTR) of their target mRNA. In addition, miRNA-mediated post-transcriptional regulation requires multiple RNA-binding proteins, which favours miRNA function in tumorigenesis (11–13).

In 2011, Salmena et al. proposed the competitive endogenous RNA (ceRNA) hypothesis, which states lncRNA can competitively bind to miRNA to regulate mRNA expression in the cytoplasm (14). Yang et al. demonstrated that LINC01133 could act as a ceRNA for miR-106a-3p to inhibit STAD progression and metastasis, and then regulate the expression of APC and the Wnt/β-catenin pathway (15). In a study by Zhang et al., MT1JP was found to regulate the progression of STAD by acting as a miR-92a-3p sponge and regulating FBXW7 expression (16).

In the present study, a lncRNA-miRNA-mRNA triple regulatory network was established in the context of STAD. After expression analysis, cellular localization analysis, correlation analysis, and survival analysis of RNAs from the triple regulatory network, a CCDC144NL-AS1-hsa-miR-145-5p-SERPINE1 ceRNA network was identified. Cox analysis showed that the CCDC144NL-AS1/SERPINE1 axis played an important role in STAD. Gene Ontology (GO) and Kyoto Encyclopedia of Genes and Genomes (KEGG) analyses were performed to understand the possible function of SERPINE1 in STAD, and methylation analysis and immune infiltration analysis were performed to investigate the potential biological function of SERPINE1 in STAD.

## Materials and Methods

### Data Preparation and Processing

RNA sequence data (lncRNA and mRNA, level3; Illumina HiSeq RNA-Seq platform), miRNA sequence data (Illumina HiSeq miRNA-Seq platform), and corresponding clinical data and sample information for STAD patients were obtained from The Cancer Genome Altas database (TCGA, https://portal.gdc.cancer.gov/). Survival information for the TCGA-STAD dataset was obtained from UCSC Xena (http://xena.ucsc.edu/). All raw RNA-seq and miRNA-seq data were normalized as fragments per kilobase per million (FPKM) and annotated based on the GENCODE database (https://www.gencodegenes.org/) and starBase database (http://starbase.sysu.edu.cn) (17, 18).

Gene expression profiles were obtained from the Gene Expression Omnibus (GEO, https://www.ncbi.nlm.nih.gov/geo/) database: GSE33335 (platform: GPL5175) and GSE66229 (platform: GPL570) were extracted to further validate the results (19). Raw data for the Affymetrix dataset were processed using the RMA algorithm for background adjustment, quantile normalization, and final summary of oligonucleotides per transcript using the median polish.

The Cancer Cell Line Encyclopedia (CCLE, https://sites.broadinstitute.org/ccle) database was used to verify the expression of SERPINE1 in STAD cell lines (20). The expression of SERPINE1 in all cell lines was determined using the “depmap portal” module and the SERPINE1 expression of different cancer cell lines was compared. The mutation status of SERPINE1 was obtained through the cBioPortal for Cancer Genomics (http://www.cbioportal.org/) (21). The alteration frequency, mutation, copy number change, and mutation site information of SERPINE1 in TCGA-STAD were observed through the “Cancer Type Summary” and “Mutation” module.

### Identification of Differentially Expressed Genes (DEGs)

For the read counts of gene expression profile data, DESeq 2 R package (http://bioconductor.org/packages/devel/bioc/html/DESeq2.html) was performed with a rigorous threshold (|log_2_-fold change (FC)| > 2.0 and FDR < 0.01) to identify the differentially expressed lncRNA, miRNA, and mRNA by comparing STAD samples and adjacent normal samples. Then, the volcano plots and heatmaps of DEGs (including DElncRNAs, DEmiRNAs, and DEmRNAs) were visualized using the ggplot2 R package (https://www.rdocumentation.org/packages/ggplot2/) in R software (https://www.r-project.org/).

### Establishment of the ceRNA Network in STAD

The lncATLAS database (https://lncatlas.crg.eu/) was used to identify the cellular localization of DElncRNAs (22). According to the hypothesis that lncRNA could indirectly regulate mRNA expression by competing with miRNA as a natural sponge in the cytoplasm, the ceRNA network was built by the following steps: (1) DIANA-LncBase v.2 (https://www.microrna.gr/LncBase) was used to predict the potential miRNAs targeted by DElncRNAs and the lncRNA-miRNA interaction pairs (23); (2) miRDB (http://mirdb.org/), TargetScan (http://www.targetscan.org) and miRTarBase (https://mirtarbase.cuhk.edu.cn/) were used to predict the target mRNAs of DEmiRNAs and construct the miRNA-mRNA interaction pairs (24–26); (3) Draw Venn Diagram (http://bioinformatics.psb.ugent.be/webtools/Venn/) was applied to compare the target miRNAs and mRNAs with DEmiRNAs and DEmRNAs, and the target miRNAs and mRNAs that overlapped with DEmiRNAs and DEmRNAs in the study were selected for the next analysis; (4) the ceRNA network was established by integrating the lncRNA-miRNA pairs and miRNA-mRNA pairs, then the Cytoscape software (http://www.cytoscape.org/) was used to visualize the lncRNA-miRNA-mRNA triple regulatory network.

### Functional Enrichment Analysis

GO and KEGG enrichment analyses were performed using the Database for Annotation, Visualization and Integrated Discovery (DAVID, https://david.ncifcrf.gov/) to investigate the potential biological processes and pathways of the ceRNA network (27). The top 200 SERPINE1-associated genes in STAD were obtained from GEPIA2 (http://gepia2.cancer-pku.cn/), GO enrichment and KEGG pathway analyses of these genes were performed using DAVID (28). All results were visualized using the R package ggplot2, and an adjusted *p* < 0.05 was considered statistically significant.

### Survival Analysis and Construction of a Specific Prognosis Model for STAD

Kaplan-Meier (K-M) survival analyses of the intersecting DElncRNAs, DEmiRNAs and DEmRNAs in the ceRNA network were performed using the R package survival (https://www.rdocumentation.org/packages/survival) to determine the relationship with the overall survival (OS) of STAD patients in the TCGA database.

Based on the high/low expression of candidate genes in STAD patients, a time-dependent receiver operating characteristic curve (ROC) was constructed. The area under the curve (AUC) was calculated to assess the predictive ability of the biomarker. Univariate and multivariate Cox regression analyses were used to analyze the association between candidate genes in the ceRNA network and OS to determine the prognostic-related biomarkers and independent prognostic factors of STAD. A *p* < 0.05 was considered statistically significant. In addition, stratification analysis was performed to determine whether the prognostic value of the biomarker remained stable in different subgroups.

### Methylation and Expression Analysis of SERPINE1

Studies have shown that DNA methylation is an obvious epigenetic mechanism that can regulate gene expression by three DNA methyltransferases (DNMT1, DNMT3A, DNMT3B) and influence cancer cell behavior. Firstly, the expression level of the three DNA methyltransferases in SERPINE1^high^ and SERPINE1^low^ groups was investigated using the TCGA-STAD dataset. In addition, the association between SERPINE1 expression and its DNA methylation status was obtained by MEXPRESS (https://mexpress.be/) (29). Finally, MethSurv (https://biit.cs.ut.ee/methsurv/) was used to perform multivariate survival analysis to evaluate the scattering of the different CpG islands (30).

### Immune Infiltrate Levels and Expression Analysis of SERPINE1

Tumor IMmune Estimation Resource (TIMER, https://cistrome.shinyapps.io/timer/), a web server for comprehensive analysis of tumor-infiltrating immune cells, was used to investigate the association between SERPINE1 expression and tumor-infiltrating immune cells (31). The correlation of SERPINE1 expression with tumor-infiltrating immune cell frequency [including B cells, CD4^+^ T cells, CD8^+^ T cells, neutrophils, macrophages, and dendritic cells (DCs)], prognostic value, and SERPINE1 copy number in STAD.

Furthermore, the correlation of SERPINE1 with the markers of 24 tumor-infiltrating immune cells [B cells, T cells, T helper cells, Tcm (T central memory), Tem (T effector memory), Th1 cells, Th2 cells, Tfh (T follicular helper), Th17 cells, Treg, CD8^+^ cells, Tgd (T gamma delta), cytotoxic cells, NK cell (natural killer cell), NK CD56dim cell, NK CD56bright cell, DC, iDC (immature DC), aDC (activated DC), pDC (plasmacytoid DC), eosinophils, macrophages, mast cells, and neutrophils] were estimated (32).

### Statistical Analysis

The obtained data were analyzed using SPSS software (SPSS, Chicago, IL, USA). The results were represented by median and 95% confidence interval (CI). Mann-Whitney test and independent t-test were utilized to calculate the difference between the two data groups. A one-way ANOVA with the Kruskal-Wallis test and the chi-square test evaluated the difference between the groups. A *p* < 0.05 was considered statistically significant.

## Results

### Identification of DEGs Between STAD Samples and Adjacent Normal Samples

**Figure 1:**
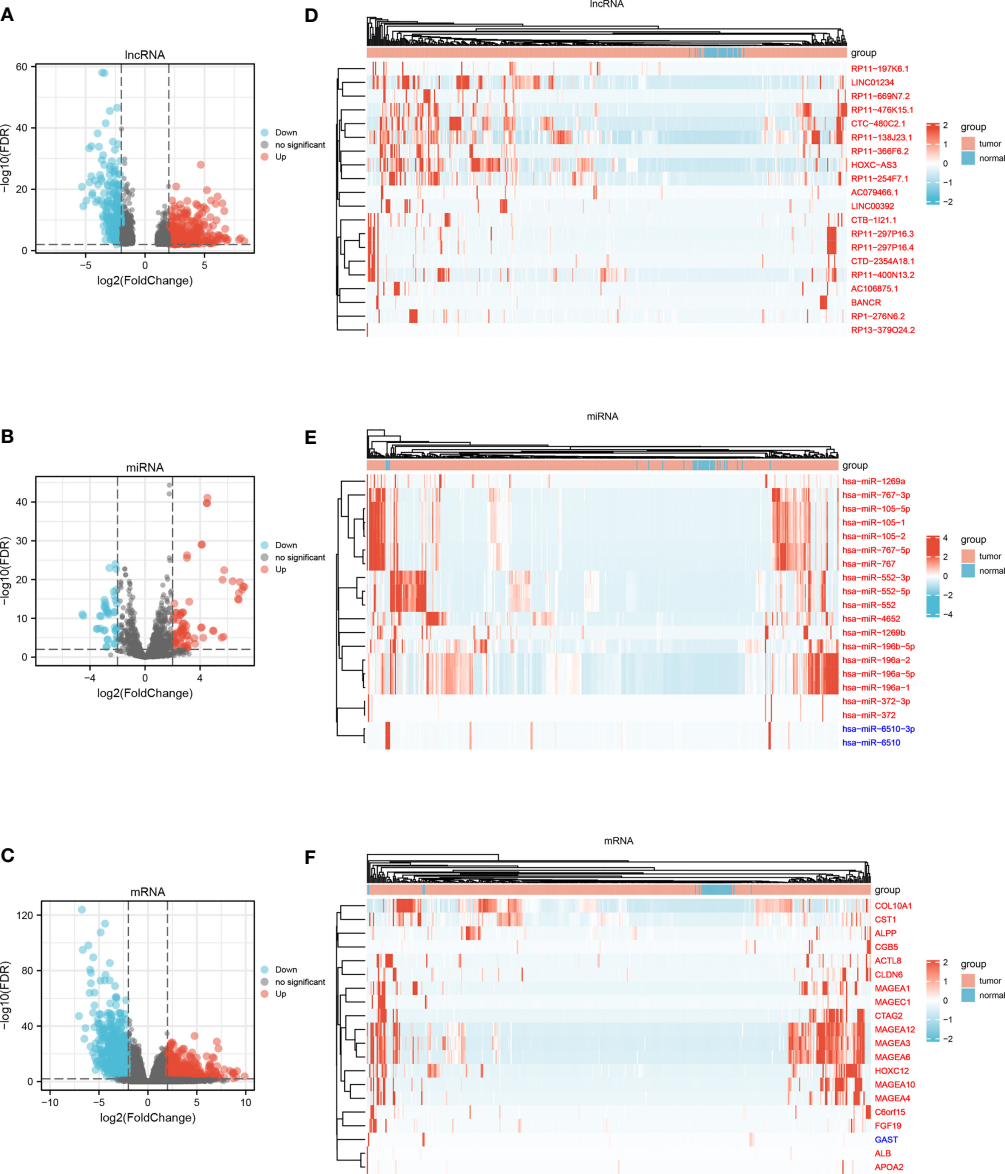
**(A)** Volcano plot of DElncRNAs. **(B) **Volcano plot of DEmiRNAs. **(C)** Volcano plot of DEmRNAs. **(D)** Heatmap of top 20 variable DElncRNAs. **(E)** Heatmap of top 20 variable DEmiRNAs. **(F)** Heatmap of top 20 variable DEmRNAs.

A total of 375 STAD samples and 32 adjacent normal samples were used to screen DElncRNAs and DEmRNAs; 446 STAD samples and 45 adjacent normal samples were used to identify DEmiRNAs. Using a rigorous threshold of |log2 FC| > 2.0 and FDR < 0.01, 565 DElncRNAs (322 upregulated and 243 downregulated), 120 DEmiRNAs (78 upregulated and 42 downregulated) and 1294 DEmRNAs (435 upregulated and 859 downregulated) were identified between STAD samples and adjacent normal samples. The volcano plots and heatmaps visually displayed the distribution of DEGs and the expression of 20 significantly variable genes in the samples from STAD and adjacent normal samples **(** **Figure 1** **)**.

### Construction of the lncRNA-miRNA-mRNA Triple Regulatory Network

**Figure 2:**
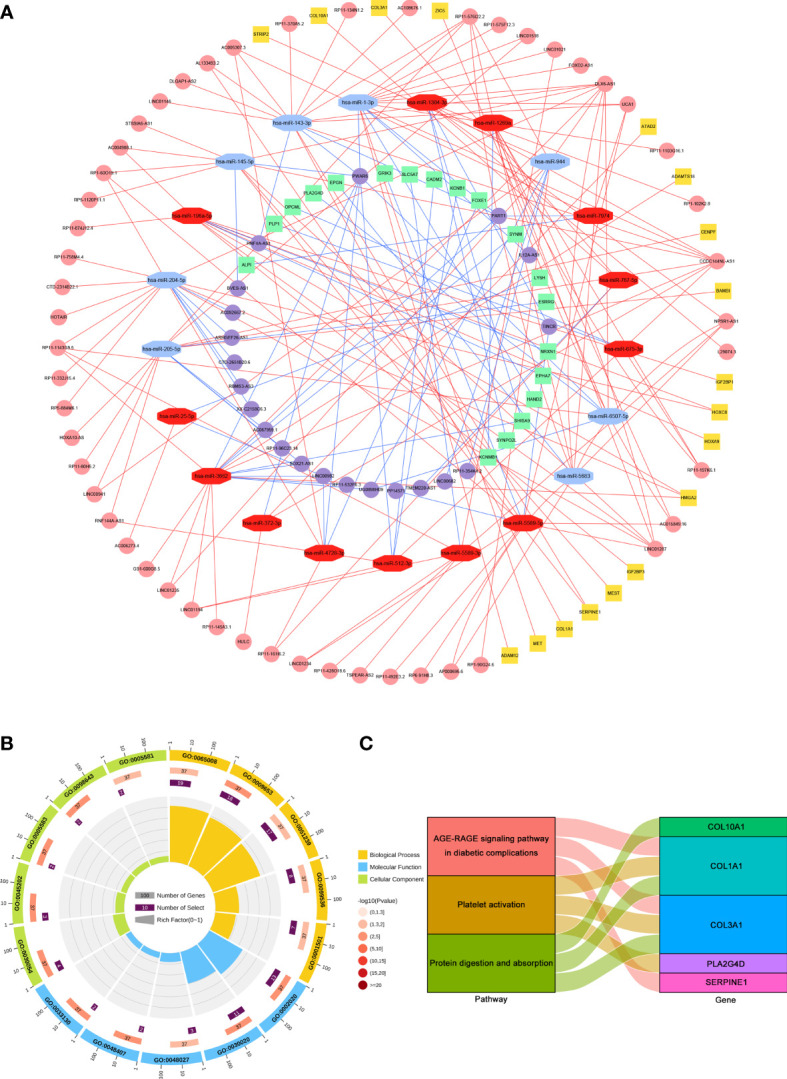
Construction and functional enrichment analysis of the lncRNA-miRNA-mRNA triple regulatory network. **(A)** The triple regulatory network in STAD. Red octagons represent upregulated miRNAs, and blue octagons represent downregulated miRNAs. Pink circles and yellow squares stand for upregulated mRNAs and lncRNAs, respectively. Purple circles and green squares present downregulated mRNAs and lncRNAs, respectively. **(B)** Circle diagram of the top 5 GO terms of BP, MF and CC. **(C)** Sankey plot of KEGG pathways analysis for the network. Rectangles on the left of the Sankey plot represent the significant pathways, while rectangles on the right represent DEmRNAs in the network.

The intracellular localization of DElncRNAs was investigated using the lncALTAS database because lncRNAs can only function as nodes of the ceRNA network in the cytoplasm. 63 DElncRNAs were located only in the cytoplasm and 86 DElncRNAs were exhibited in the nuclear and cytoplasm. First, the remaining DElncRNAs were entered into the DIANA-LncBase v.2 database to identify the potential miRNAs targeting lncRNAs. The miRNAs that matched with DElncRNAs were selected to predict mRNAs. Then, miRDB, TargetScan and miRTarBase were utilized to screen the downstream target mRNAs. The potential mRNAs shared only by all three databases were selected to increase the reliability of the prediction. Finally, a total of 72 lncRNAs (51 upregulated and 21 downregulated), 21miRNAs (13 upregulated and 8 downregulated), and 37 mRNAs (18 upregulated and 19 downregulated) were included in the STAD-associated lncRNA-miRNA-mRNA triple regulatory network using Cytoscape software **(** **Supplementary Table S1** and **Figure 2A** **)**.

To further explore the biological functions and pathways associated with the triple regulatory network, GO and KEGG enrichment analysis was performed *via* DAVID. The GO terms and KEGG pathways were considered statistically significant at an adjusted *p* < 0.05. The results of GO analysis showed that DElncRNAs involved in the network were mainly enriched in “Regulation of biological quality” [biological process (BP), GO: 0065008], “Protease binding” [molecular function (MF), GO: 0002020] and “Cell junction” [cellular component (CC), GO: 0030054] **(** **Figure 2B** **)**. The results of KEGG analysis showed that DElncRNAs involved in the network were particularly enriched in “AGE-RAGE signaling pathway in diabetic complications” (hsa04933), “Protein digestion and absorption” (hsa04974) and “Platelet activation” (hsa04611) **(** **Figure 2C** **)**.

### Survival Analysis of the Triple Regulatory Network-Associated Genes

**Figure 3:**
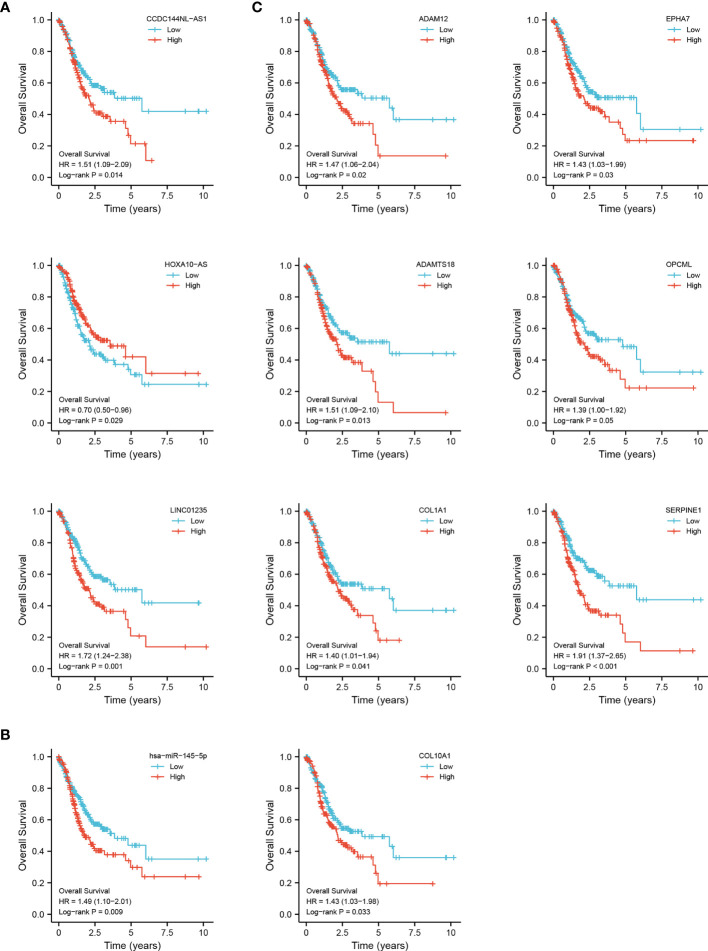
Overall survival analysis for the DEGs in the triple regulatory network. The high-expression and low-expression values of 11 survival-related genes were compared by a K-M survival curve for TCGA STAD patient cohort. **(A)** 3 DElncRNAs. **(B)** 1 DEmiRNA. **(C)** 7 DEmRNAs. The horizontal axis indicates the overall survival time in years, and the vertical axis represents the survival rate.

To identify the potential DEGs that strongly correlate with the prognostic characteristics of STAD patients, K-M survival analysis and log-rank tests were performed for each gene to evaluate the contribution of 72 DElncRNAs, 21 DEmiRNAs, and 37 DEmiRNAs. Overall, 3 DElncRNAs (CCDC144NL-AS1, HOXA10-AS, and LINC01235), 1 DEmiRNA (has-miR-145-5p) and 7 DEmRNAs (ADAM12, ADAMTS18, COL1A1, COL10A1, EPHA7, OPCML, and SERPINE1) were associated with prognosis (*p* < 0.05) **(** **Figure 3** **)**.

### Construction and Validation of the ceRNA Network and Selection of a Model With STAD-Specific Prognostic Value

**Figure 4:**
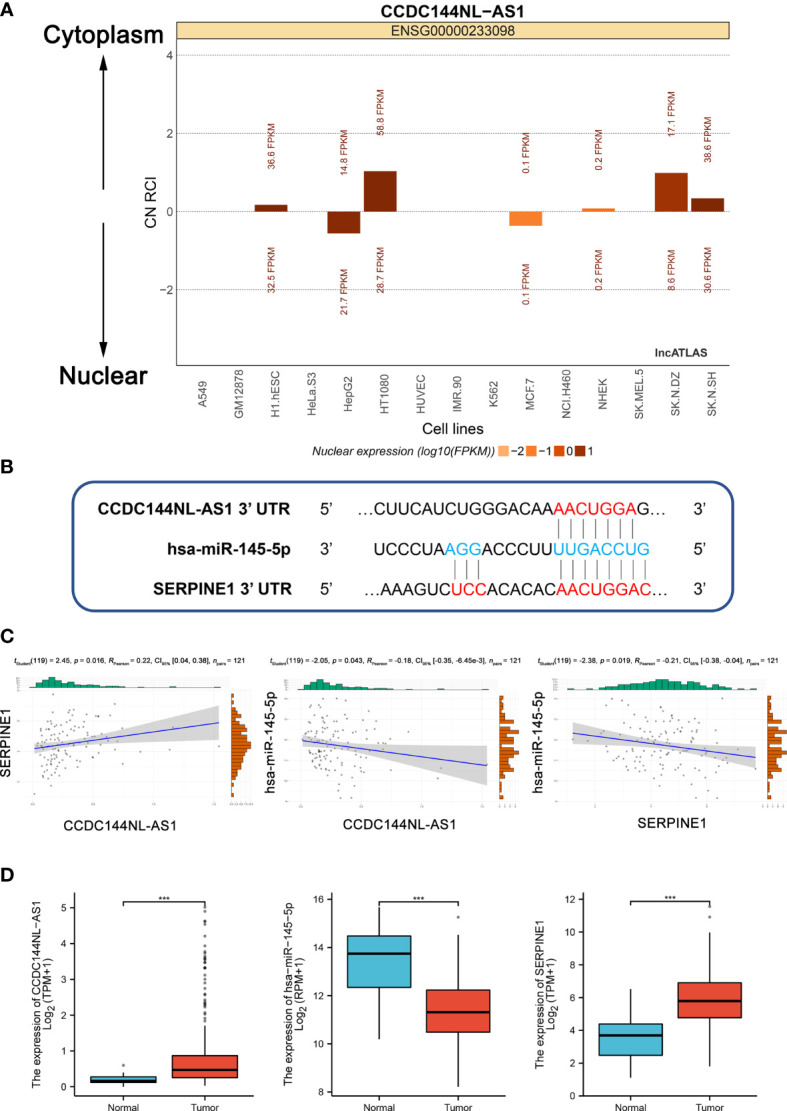
Construction and correlation analysis of the ceRNA network. **(A)** The cellular localization for CCDC144NL-AS1 was predicted using lncALTAS. **(B)** Base pairing between hsa-miR-145-5p and the target site in the CCDC144NL-AS1 and SERPINE1 3’ UTR predicted by DIANA-LncBase v.2 and TargetScan, respectively. **(C)** Correlation analysis among CCDC144NL-AS1, hsa-miR-145-5p and SERPINE1 in STAD. **(D)** The expression levels of CCDC144NL-AS1, hsa-miR-145-5p and SERPINE1. ***p <0.001.

According to ceRNA mechanism theory, lncRNAs positively regulate mRNA expression by directly interacting with miRNA. As mentioned above, CCDC144NL-AS1 was mainly located in the cytoplasm, and it may act as a ceRNA to improve the expression of SERPINE1 through sponging hsa-miR-145-5p **(** **Figure 4A** **)**. A CCDC144NL-AS1/hsa-miR-145-5p/SERPINE1 network was established. The target sites in the 3’ UTRs of CCDC144NL-AS2 and SERPINE1 were predicted by DIANA-LncBase v.2 and TargetScan, respectively, for pairing with has-miR-145-5p **(** **Figure 4B** **)**.

Patients with complete CCDC144NL-AS1, hsa-miR-145-5p and SERPINE1 expression profile data and clinical information, OS > 30 days were selected for molecular correlation analysis. The results showed a positive relationship between CCDC144NL-AS1 expression and SERPINE1 expression (*R*_Pearson_ = 0.22), a negative relationship between hsa-miR-145-5p expression and CCDC144NL-AS1 expression (*R*_Pearson_ = -0.18), and a negative relationship between hsa-miR-145-5p expression and SERPINE1 expression (*R*_Pearson_ = -0.21) **(** **Supplementary Table S2** and **Figure 4C** **)**. The expression levels of CCDC144NL-AS1 and SERPINE1 were higher in tumor tissues than in adjacent normal tissues, whereas the expression of has-miR-145-5p was opposite (**Figure 4D**). Therefore, the CCDC144NL-AS1/SERPINE1 axis in ceRNA network was considered as a potential prognostic model for the next step of the analysis.

### Clinical Relevance of the CCDC144NL-AS1/SERPINE1 Axis in STAD Patients

To determine whether the expression levels of CCDC144NL-AS1 and SERPINE1 are influenced by clinical characteristics, the correlation of CCDC144NL-AS1 expression and SERPINE1 expression with clinical factors were examined. The results indicated that CCDC144NL-AS1 expression was positively correlated with T stage (*p* = 0.011) and SERPINE1 expression was positively correlated with histological grade (*p* = 0.013). The expression levels of CCDC144NL-AS1 and SERPINE1 were not associated with age, gender, Pathologic stage, N stage and M stage **(** **Supplementary Table S3** **)**.

### Construction and Validation of a CCDC144NL-AS1/SERPINE1 Axis Prognostic Model

**Figure 5:**
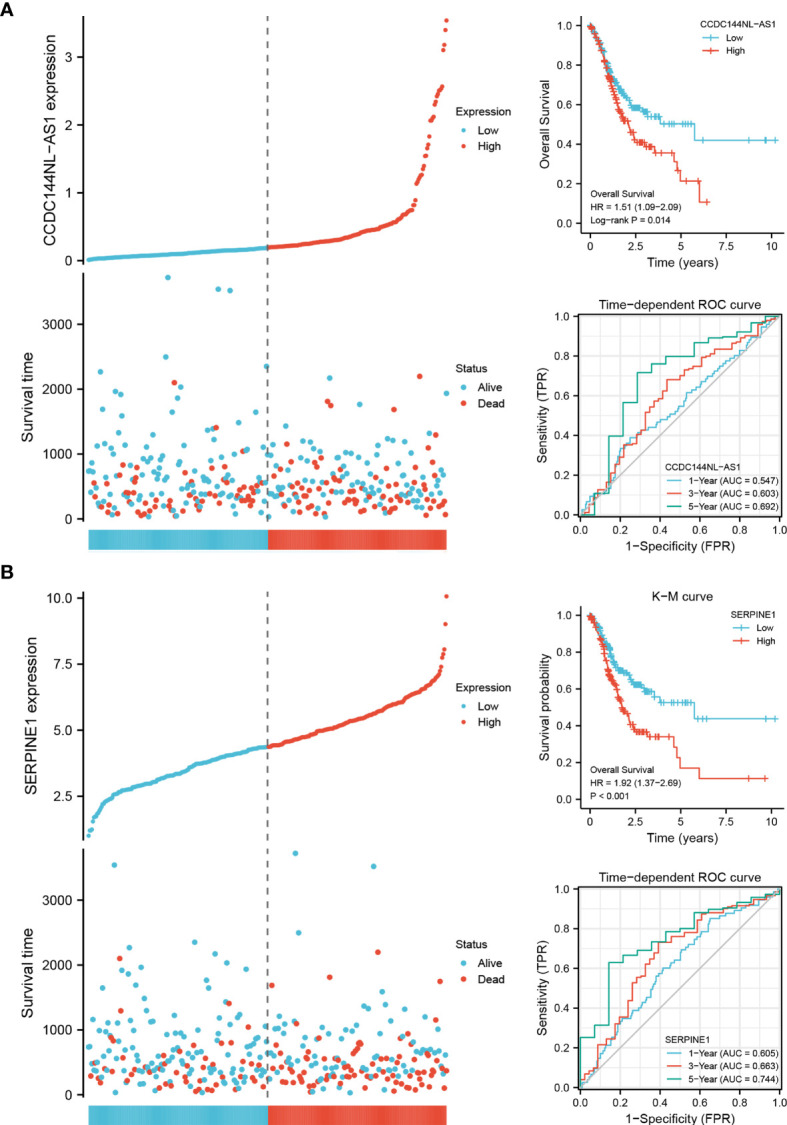
The prognostic biomarkers of CCDC144NL-AS1 and SERPINE1. **(A)** Expression system, K-M survival analysis and time-dependent ROC curve analysis of CCDC144NL-AS1. **(B)** Expression system, K-M survival analysis and time-dependent ROC curve analysis of SERPINE1.

CCDC144NL-AS1 and SERPINE1 were the candidate genes to construct a specific prognostic biomarker for STAD. The expression of the two genes was divided into groups based on the median, and K-M survival analysis showed that the patients with high-expression had a shorter OS than the patients with low-expression. High-expression of CCDC144NL-AS1 and SERPINE1 were the high-risk factors for the prognosis of STAD patients [CCDC144NL-AS1: hazard ratio (HR) = 1.51, SERPINE1: HR = 1.92]. Time-dependent ROC curve analysis confirmed the great prognostic value of CCDC144NL-AS1 and SERPINE1 **(** **Figure 5** **)**. The AUC of CCDC144NL-AS1 for OS was 0.547 at 1 year, 0.603 at 3 years and 0.692 at 5 years, and the AUC of SERPINE1 for OS was 0.605 at 1 year, 0.663 at 3 years and 0.744 at 5 years. The results showed that SERPINE1 had a better prognostic value.

**Figure 6:**
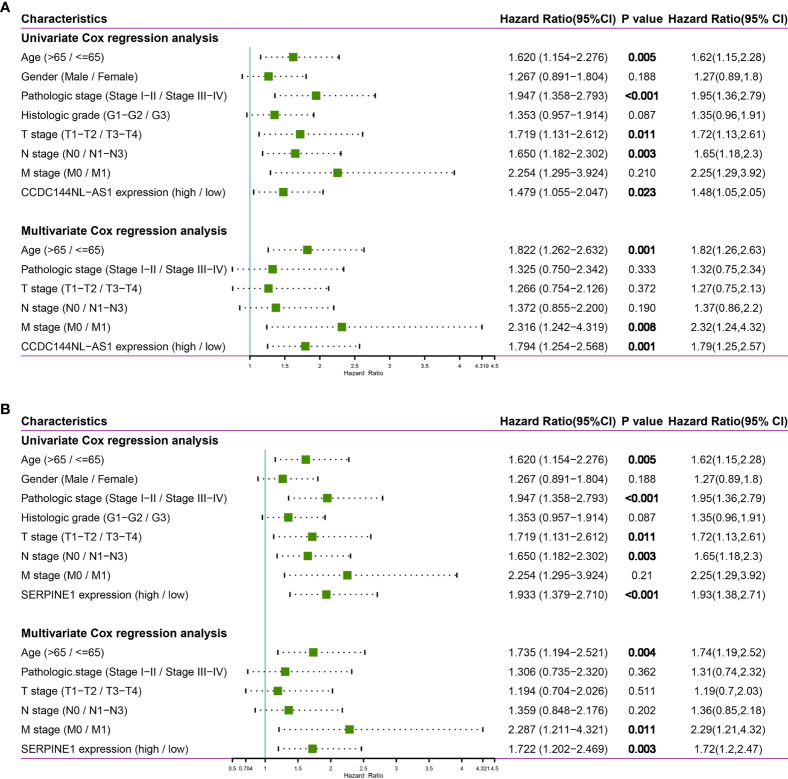
Univariate and multivariate Cox regression analysis of CCDC144NL-AS1 and SERPINE1. **(A)** The forest plot of CCDC144NL-AS1. **(B)** The forest plot of SERPINE1.

In order to further analyze the significance of clinical characteristics on prognosis, univariate and multivariate Cox regression analysis were performed to determine OS-related characteristics. In the univariate Cox regression analysis models of CCDC144NL-AS1 and SERPINE1, some prognostic factors (age, pathologic stage and TNM stage) were closely associated with OS (*p* < 0.05) in STAD patients in TCGA cohorts. Importantly, overexpression levels of CCDC144NL-AS1 (HR = 1.479, *p* = 0.023) and SERPINE1 (HR = 1.933, *p* < 0.001) were significantly correlated with poor prognosis. In the multivariate Cox regression analysis of CCDC144NL-AS1 and SERPINE1, age (CCDC144NL-AS1: HR = 1.822, *p* = 0.001; SERPINE1: HR = 1.735, p = 0.004), M stage (CCDC144NL-AS1: HR = 2.316, *p* = 0.008; SERPINE1: HR = 2.287, p = 0.011), CCDC144NL-AS1^high^ expression (HR = 1.794, *p* = 0.001) and SERPINE1^high^ expression (HR = 1.722, p = 0.003) were closely associated with OS in STAD patients **(** **Figure 6** **)**. Thus, elevated CCDC144NL-AS1 and SERPINE1 levels were related with poor prognosis and could become independent prognostic factors for STAD patients.

**Figure 7:**
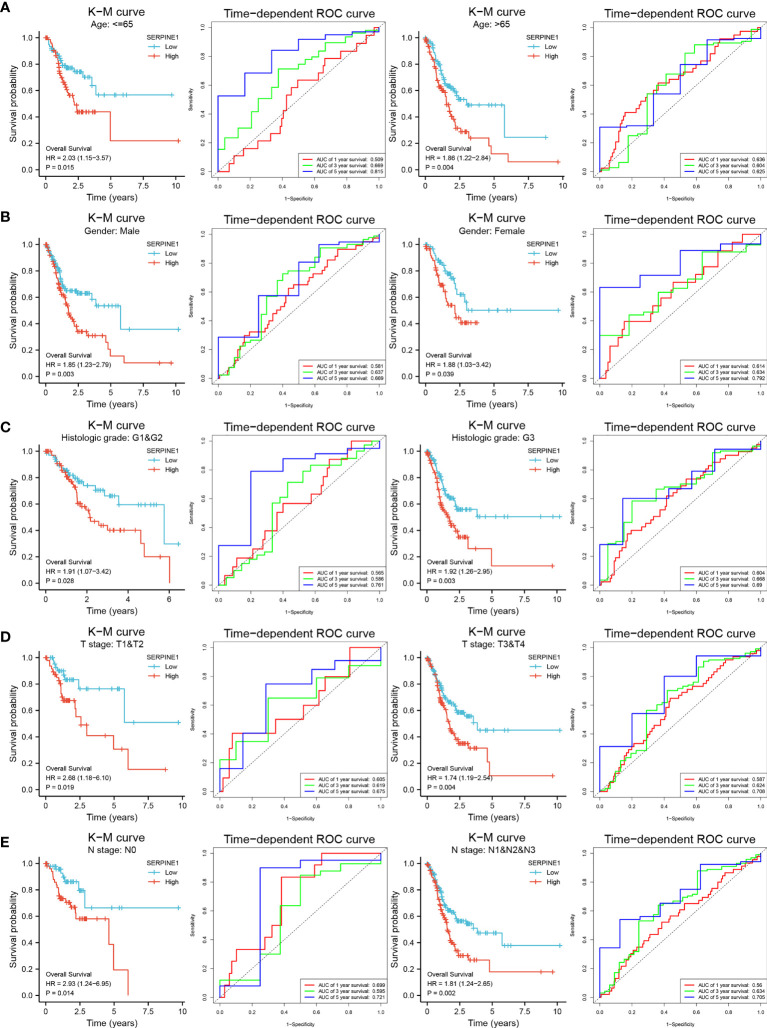
Stratification analysis of SERPINE1 biomarker. K-M curves and time-dependent ROC curves illustrate the prognostic value of the SERPINE1 biomarker based on the stratification of different clinical features. **(A)** Age. **(B)** Gender. **(C)** Histologic grade. **(D)** T stage. **(E)** N stage.

Stratification analysis was utilized to investigate whether the prognostic value of the biomarker remained stable in different subgroups. Therefore, patients in the TCGA-STAD cohort were divided into two groups according to the clinical information. After stratification analysis, SERPINE1 remained a stable and great predictive ability for STAD patients in different subgroups (age, gender, histologic grade, T stage and N stage) **(** **Figure 7** **)**, but CCDC144NL-AS1 did not apply to subgroup prediction. Thus, SERPINE1 may become a better prognostic biomarker for STAD patients.

### Validation of SERPINE1 Abnormally High Expression

**Figure 8:**
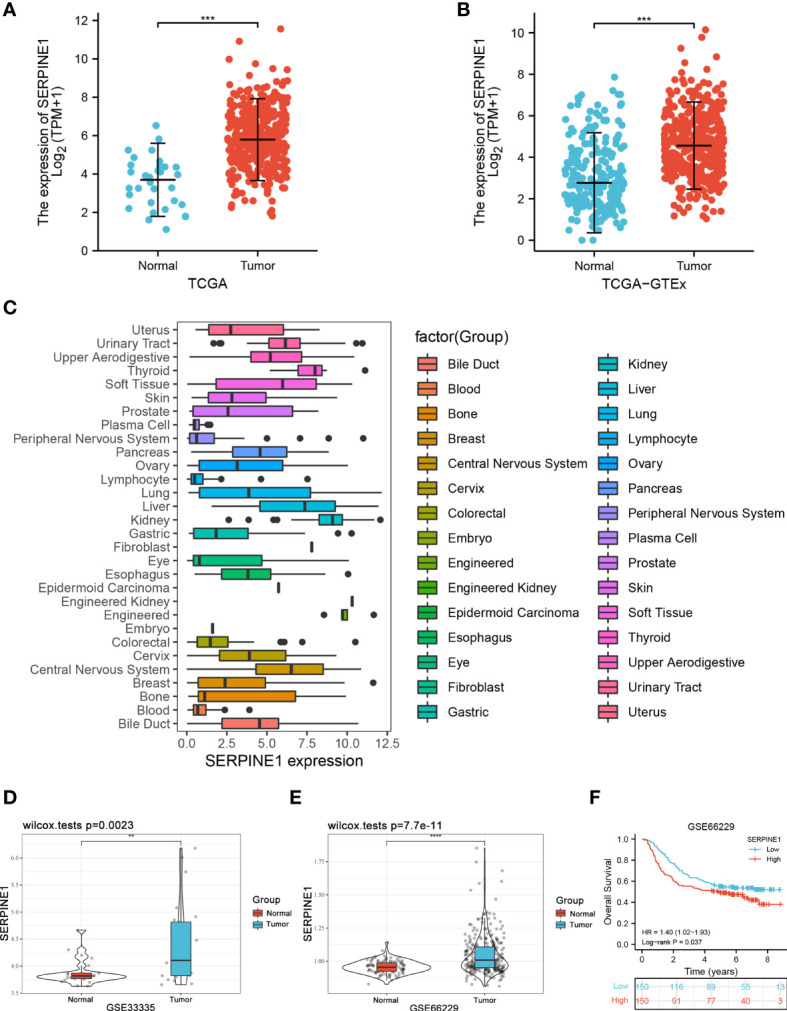
Validation of SERPINE1 expression. **(A)** The expression of SERPINE1 in TCGA-STAD cohort of 32 adjacent normal samples and 375 tumor samples. **(B)** The expression of SERPINE1 in TCGA-STAD cohort and GTEx cohorts of 206 normal samples and 375 tumor samples. **(C)** Expression distribution of SERPINE1 in pan-cancer cell lines. **(D)** The expression of SERPINE1 in the GSE33335 STAD cohort of 25 paired STAD samples. **(E)** The expression of SERPINE1 in the GSE66229 STAD cohort of 100 normal samples and 300 tumor samples. **(F)** Survival analysis of GSE66229. **p < 0.01, ***p < 0.001, ****p < 0.0001.

To better understand the role of the CCDC144NL-AS1/SERPINE1 axis in STAD, SERPINE1 is analyzed in detail. First, using STAD patients in the TCGA cohorts and the normal tissues in the GTEx cohorts (174 normal samples, 32 adjacent normal samples and 375 tumor samples), SERPINE1 was found to have high expression in the tumor group **(** **Figures 8A, B** **)**. Then, the CCLE database was used to explore the expression of SERPINE1 in STAD cell lines. The result indicated an overexpression of SERPINE1 in STAD cell lines **(** **Figure 8C** **)**. In addition, the differential analysis of SERPINE1 was performed in STAD patients from GSE33335 (25 paired STAD samples) and GSE66229 (100 normal samples and 300 STAD samples) in GEO database to further verify the expression of SERPINE1. Survival analysis of GSE66229 showed that abnormally high expression of SERPINE1 was a risk factor for OS in STAD patients (HR = 1.40, *p* = 0.037) **(** **Figures 8D–F** **)**. The results revealed that the expression of SERPINE1 in the tissues of STAD was significantly higher in tumor tissues than in normal tissues and adjacent tissues, which was consistent with the results of TCGA, GEO, and CCLE database.

Considering that a genetic mutation could be a potential mechanism promoting the abnormal overexpression of SERPINE1, the following analysis was performed. An OncoPrint plot indicated the amplification of SERPINE1 in the TCGA-STAD dataset. However, there was no significant correlation between SERPINE1 expression and copy number in the STAD samples **(** **Supplementary Figure S1** **)**. Therefore, these results showed that the abnormal overexpression of SERPINE1 was not correlated with the genetic mutation in STAD.

### Relationship Between Methylation and Expression of SERPINE1

**Figure 9:**
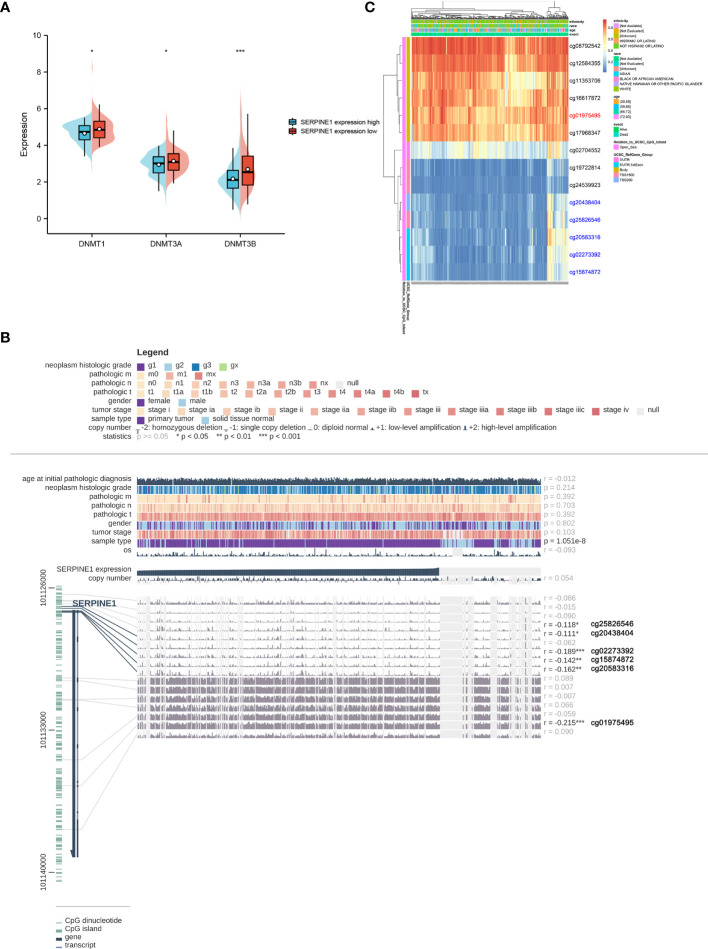
Methylation analysis of SERPINE1. **(A)** Differential expression of three DNA methyltransferases (DNMT1, DNMT3A and DNMT3B). **(B)** The methylation site of SERPINE1 DNA sequence association with gene expression was visualized using MEXPRESS. The blue line illustrates the expression of SERPINE1 in the center of the plot. Pearson’s correlation coefficients and *p* values for methylation sites and query gene expression are shown on the right side. **(C)** Differently methylated regions associated with SERPINE1 were presented by heatmap using MethSurv.

To further clarify the abnormal mechanism of SERPINE1 upregulation in STAD tissues, the correlation between the expression levels of SERPINE1 and its methylation status was explored by various methods. First, the differential expression of three DNA methyltransferases (DNMT1, DNMT3A and DNMT3B) in the SERPINE1^high^ and SERPINE1^low^ groups of TCGA-STAD was analyzed. The results demonstrated that the expression levels of DNMT1, DNMT3A and DNMT3B were significantly lower in the SERPINE1^high^ group than in the SERPINE1^low^ group **(** **Figure 9A** **)**. Second, six methylation sites (cg25826546, cg20438404, cg02273392, cg15874872, cg20583316 and cg01975495) in the DNA sequences of SERPINE1 were found to be negatively correlated with its expression **(** **Figure 9B** **)**. Finally, the differential methylation regions related to SERPINE1 were shown as heatmaps **(** **Figure 9C** **)**.

### Correlation Between Immune Infiltration and Expression of SERPINE1 in STAD

**Figure 10:**
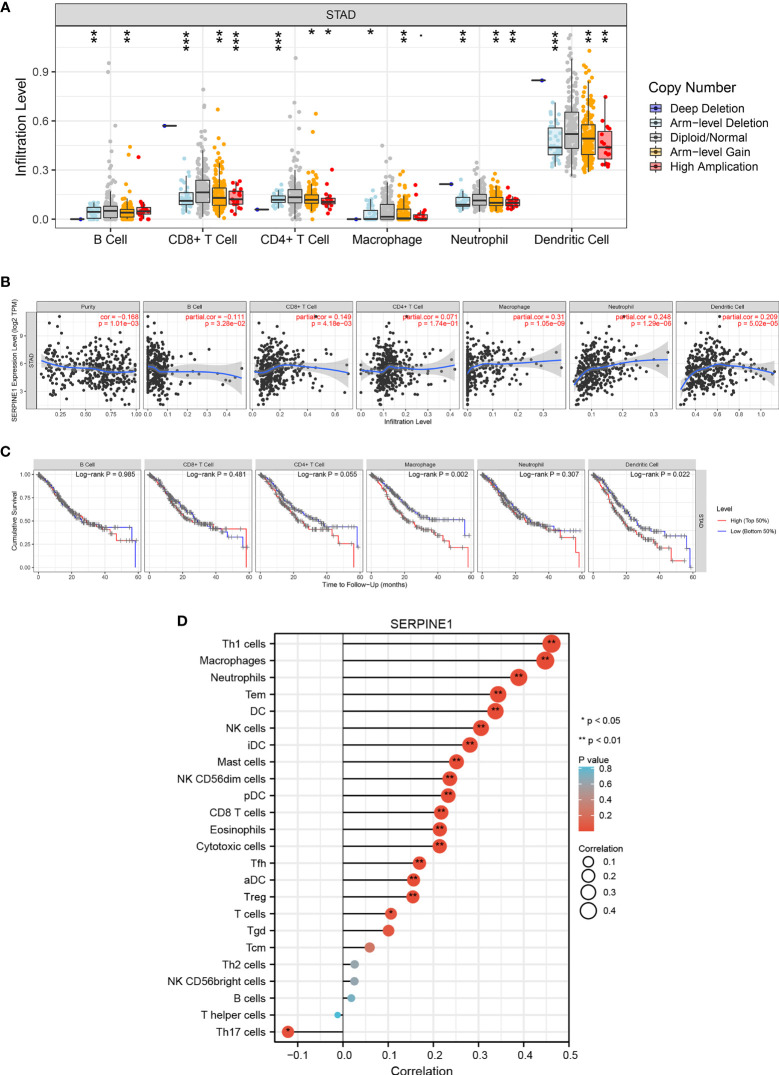
Correlation analysis of SERPINE1 expression and immune infiltration in STAD. **(A)** Association between SERPINE1 gene copy number and immune cell infiltration levels in STAD cohorts. **(B)** Correlation of SERPINE1 expression with immune infiltration level in STAD. **(C)** K-M plots of immune infiltration and overall survival rate of STAD. **(D)** Lollipop graphs of correlation between SERPINE1 and biomarkers of 24 immune cells. *p < 0.05, **p < 0.01, ***p < 0.001.

To evaluate the potential relationship between SERPINE1 expression and STAD immune infiltration, the following analyze were performed using TIMER. First, analysis of the “SCNA” module revealed that different levels of immune cell infiltration appeared to be associated with altered SERPINE1 gene copy numbers, including B cells, CD8^+^ T cells, CD4^+^ T cells, macrophages, neutrophils, and dendritic cells in STAD **(** **Figure 10A** **)**. Analysis of “Gene” module indicated that SERPINE1 expression was significantly negatively correlated with tumor purity and the level of infiltration of B cells, and significantly positively associated with the level of infiltration of CD8^+^ cells, macrophages, neutrophils and DCs in STAD **(** **Figure 10B** **)**. Finally, the effects of immune infiltration on the clinical prognosis of STAD patients were further evaluated. The results showed that the high levels of macrophages and DCs were related to the poor prognosis of STAD patients with survival time less than 60 months **(** **Figure 10C** **)**. The above results suggested that the CCDC144NL-AS1/SERPINE1 axis might affect STAD and clinical prognosis by regulating the level of tumor-infiltrating immune cells.

To further verify the relationship between SERPINE1 and a variety of immune-infiltrating cells, the associations between SERPINE1 and immune marker sets of 24 immune cells were explored. The expression of 17 markers (Th1 cells, macrophages, neutrophils, Tem, DC, NK cells, iDC, mast cells, NK CD56dim cells, pDC, CD8^+^ T cells, eosinophils, cytotoxic cells, Tfh, aDC, Treg, and T cells) had significant positive correlations with SERPINE1 expression in STAD, and Th17 cells expression was significantly negatively associated with SERPINE1 expression in STAD **(** **Figure 10D** and **Supplementary Table S4** **)**. These results suggest that tumor-infiltrating immune cells may play an important role in the clinical outcome of the CCDC144NL-AS1/SERPINE1 axis in STAD.

### SERPINE1-Related Functional Enrichment Analysis in STAD

**Figure 11:**
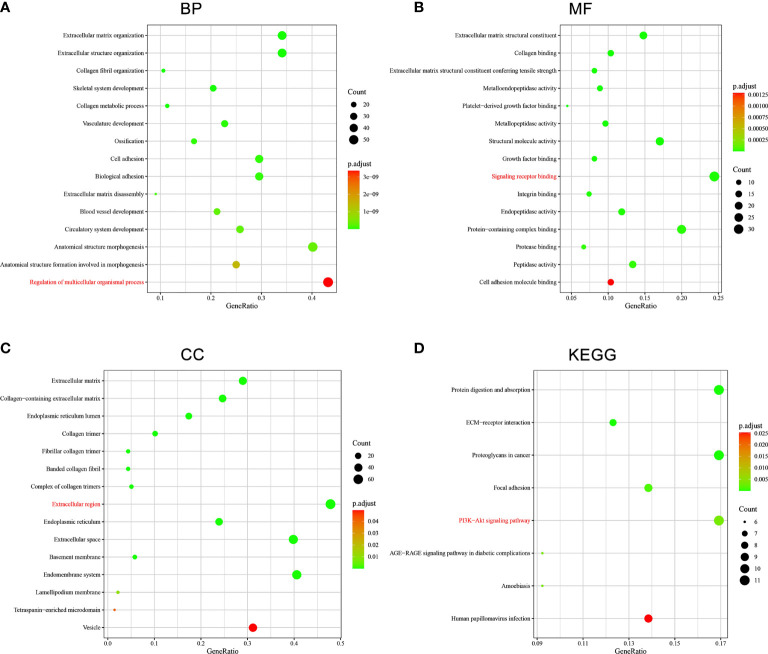
Functional enrichment analysis of SERPINE1-related genes in STAD. **(A)** GO : BP. **(B)** GO : MF. **(C)** GO : CC. **(D)** KEGG. The terms in red were the mainly enriched GO terms and KEGG pathway.

GO and KEGG pathway enrichment analysis of the top 200 correlated genes of SERPINE1 were performed to explore further possible function of SERPINE1 in STAD **(** **Figure 11** **)**. The KEGG pathway enrichment term related to SERPINE1 was “PI3K-AKT signaling pathway” (hsa04151). The GO enrichment terms associated with SERPINE1 were mainly enriched in “Regulation of multicellular organismal process” (BP, GO:0051239), “Signaling receptor binding” (MF, GO:0005102) and “Extracellular region” (CC, GO:0005576).

## Discussion

STAD is one of the most common cancers with poor prognosis and high mortality. It is difficult to detect STAD in early stages, resulting in delayed diagnosis of STAD and poor survival (33, 34). Radiotherapy, chemotherapy, surgery, and combination therapy are commonly used to treat STAD (35). However, due to internal metastasis and tumor changes, heterogeneity of different patients, and side effects of radiotherapy and chemotherapy, patients’ options are limited in clinical practice (36). Clarifying the molecular mechanisms and processes of STAD pathogenesis and determining promising biomarkers is crucial for identifying new therapeutic targets and improving patients’ prognosis. The ceRNA regulatory network has been reported to be involved in the occurrence and development of many human cancers, including lung cancer, liver cancer, and pancreatic cancer (37–39). To our knowledge, few studies have focused on a systematic ceRNA regulatory network to predict the prognosis of STAD. In this study, a lncRNA-miRNA-mRNA triple regulatory network was constructed in STAD. Finally, CCDC144NL-AS1-hsa-miR-145-5p-SERPINE1 ceRNA network was determined to be associated with the prognosis of STAD.

The literature search revealed that CCDC144NL-AS1, hsa-miR-145-5p and SERPINE1 had been studied for their role in cancer or their association with cancer. Some studies suggest that CCDC144NL-AS1 could promote the development of hepatocellular carcinoma, non-small cell lung cancer and osteosarcoma by acting as a molecular sponge for various miRNA (40–42). Fan et al. performed bioinformatics analysis, *in vivo* and *in vitro* experiments and concluded that CCDC144NL-AS1 in GC can promote cell proliferation, invasion, migration and inhibit cell apoptosis (43). The study by Ozen et al. showed that overexpression of hsa-miR-145-5p inhibited the proliferation of prostate cancer cells and reduced SOX2 expression (44). Another study demonstrated that downregulation of hsa-miR-145-5p in cancer cells might contribute to the development of ovarian cancer (45). Zhang’s research suggested that hsa-miR-145-5p can bind to circDUSP16 and attenuate the promotion of GC (46). Some relevant studies have shown that high expression of SERPINE1 is a poor prognostic marker for breast cancer and pancreatic ductal adenocarcinoma (47, 48). Yang et al. explored the function of SERPINE1 on STAD cells and found that over-expression of SERPINE1 contributes to the cells proliferation, invasion, and migration of STAD cells (49). The above studies suggest that CCDC144NL-AS1 and SERPINE1 are oncogenes, and hsa-miR-145-5p is an anti-oncogene, which is consistent with this study. Hsa-miR-145-5p can inhibit translation of SERPINE1 or leads to degradation of SERPINE1 by combining SERPINE1 to microRNA response elements (MREs) on SERPINE1. CCDC144NL-AS1 has similar MREs to SERPINE1, implying that CCDC144NL-AS1 can also bind to hsa-miR-145-5p. Based on the ceRNA regulatory mechanism, it is speculated that disrupting the balance of the CCDC144NL-AS1-hsa-miR-145-5p-SERPINE1 axis will lead to the occurrence and development of STAD.

In this study, the expression of CCDC144NL-AS1 and SERPINE1 was significantly higher in STAD than in normal tissues. The high expression of CCDC144NL-AS1 and SERPINE1 was associated with short OS in survival analysis. Some studies had shown that DNA methylation plays an important role in regulating gene expression, which may cause the abnormal overexpression of SERPINE1 (50, 51). The DNA methylation patterns that could lead to abnormal expression of SERPINE1 in STAD have been explored using some relevant websites and databases. It was found that some methylation sites were negatively correlated with the prognosis of STAD patients. The results of association between SERPINE1 expression and genome-wide methylation showed that all hypermethylation sites were located in the Body region and more hypomethylated sites were located in the TSS1500, TSS200, 5’ UTR, 1st Exon regions. The methylation sites cg25826546, cg20438404, cg02273392, cg15874872 and cg20583316 in the DNA sequences of SERPINE1 were hypomethylated in STAD compared to adjacent normal tissues, which was consistent with the results of DNA methyltransferase analysis of SERPINE1 in STAD. These findings suggest that low methylation levels of cg25826546, cg20438404, cg02273392, cg15874872 and cg20583316 might promote high expression of SERPINE1, leading to poor prognosis in STAD patients.

Previous studies have reported that the prognosis of patients could be affected by immune infiltration (52, 53). In the present study, SERPINE1 gene copy numbers were negatively related to different levels of immune infiltration (CD8^+^ cells, CD4^+^ cells, macrophages, neutrophils, and DCs). It was also found that SERPINE1expression was highly associated with immune infiltration of STAD. In addition, some tumor-infiltrating immune cells were significantly correlated with the prognosis of STAD patients (54–56). Notably positive correlations were found between SERPINE1 expression and some immune marker sets in Th1 cells, macrophages and neutrophils in STAD. The above results suggest that these differences induced by the CCDC144NL-AS1/SERPINE1 axis might affect the changes in the tumor immunological microenvironment and the development of STAD.

Although ceRNA-based CCDC144NL-AS1/SERPINE1 axis, which appeared to be a potential prognostic biomarker for clinical application, has been established, several limitations should be noted. The obtained binding affinity of lncRNA, miRNA and mRNA from the database needed further experimental investigation. Also, the role and mechanism of CCDC144NL-AS/SERPINE1 axis at STAD should be further investigated experimentally.

## Conclusion

In summary, a ceRNA overexpression network (CCDC144NL-AS1-hsa-miR-145-5p-SERPINE1) associated with the prognosis of STAD was constructed, which is more conducive to understanding the correlation between lncRNA-miRNA-mRNA. Moreover, ceRNA-based CCDC144NL-AS1/SERPINE1 axis might be a new important prognostic factor for STAD, and the prognostic biomarker helped to explore the pathogenesis of STAD.

## Data Availability Statement

The original contributions presented in the study are included in the article/**Supplementary Material**. Further inquiries can be directed to the corresponding author.

## Author Contributions

ZH conceived, designed and performed the research and wrote the paper. XL provided guidance in editing codes for bioinformatics analysis and substantive suggestions for revising the manuscript. WZ, LY, and ZJ provided useful suggestions in methodology. CW and SL performed data analysis. JZ, ZW, YT, and XF provided suggestions for the manuscript. SA refined and polished the language of the manuscript. JW supervised the research. All authors contributed to the article and approved the submitted version.

## Funding

The study was financially supported by the National Natural Science Foundation of China (Grant nos. 82074284).

## Conflict of Interest

The authors declare that the research was conducted in the absence of any commercial or financial relationships that could be construed as a potential conflict of interest.

## Publisher’s Note

All claims expressed in this article are solely those of the authors and do not necessarily represent those of their affiliated organizations, or those of the publisher, the editors and the reviewers. Any product that may be evaluated in this article, or claim that may be made by its manufacturer, is not guaranteed or endorsed by the publisher.

## Supplementary Material

Supplementary Table 1 Information of STAD-associated lncRNA-miRNA-mRNA triple regulatory network.

Supplementary Table 2 Univariate analysis of overall survival in STAD patients stratified based on clinical characteristics.

Supplementary Table 3 Correlation analysis between SERPINE1 and biomarkers of immune cells.

Supplementary Figure 1 The mutation status of SERPINE1 in STAD. **(A)** The distribution of SERPINE1 genomic alterations in the TCGA-STAD dataset is shown on the cBioPortal OncoPrint plot. **(B)** The correlation analysis between SERPINE1 copy number and mRNA expression showed in the box-point plot. **(C)** The correlation analysis between SERPINE1 copy number and mRNA expression showed in the correlation plot.

## Abbreviations

AUC, the area under the curve; ceRNA, competitive endogenous RNA; DEGs, differentially expressed genes; GC, gastric carcinoma; GO, Gene Ontology; HR, hazard ratio; KEGG, Kyoto Encyclopedia of Genes and Genomes; lncRNA, long noncoding RNA; miRNA, microRNA; MREs, microRNA response elements; OS, overall survival; STAD, stomach adenocarcinoma; UTR, untranslated region.
