# Supplementary material for: A New Strategy to Identify ceRNA-Based CCDC144NL-AS1/SERPINE1 Regulatory Axis as a Novel Prognostic Biomarker for Stomach Adenocarcinoma via High Throughput Transcriptome Data Mining and Computational Verification
Source: Front Oncol. 2022 Jan 27;11:802727. doi: 10.3389/fonc.2021.802727 (PMC8828946; doi:10.3389/fonc.2021.802727)
Supplement: Supplementary Table 1 — Information of STAD-associated lncRNA-miRNA-mRNA triple regulatory network. [file DataSheet_1.docx]

**Table S1. Information of STAD-associated lncRNA-miRNA-mRNA triple regulatory network.**

| lncRNA | miRNA | mRNA |
| --- | --- | --- |
| AC004988.1 | hsa-miR-196a-5p | EPHA7 |
| AC004988.1 | hsa-miR-196a-5p | HMGA2 |
| AC004988.1 | hsa-miR-196a-5p | HOXA9 |
| AC004988.1 | hsa-miR-196a-5p | HOXC8 |
| AC004988.1 | hsa-miR-196a-5p | IGF2BP1 |
| AC004988.1 | hsa-miR-145-5p | MEST |
| AC004988.1 | hsa-miR-196a-5p | NRXN1 |
| AC004988.1 | hsa-miR-145-5p | SERPINE1 |
| AC005307.3 | hsa-miR-7974 | ALPI |
| AC005307.3 | hsa-miR-143-3p | COL1A1 |
| AC005307.3 | hsa-miR-4728-3p | FOXE1 |
| AC005307.3 | hsa-miR-143-3p | SERPINE1 |
| AC006273.4 | hsa-miR-25-5p | LY6H |
| AC015849.16 | hsa-miR-5589-5p | GRIK3 |
| AC015849.16 | hsa-miR-1269a | KCNMB1 |
| AC015849.16 | hsa-miR-1269a | KCNMB1 |
| AC067959.1 | hsa-miR-205-5p | BAMBI |
| AC067959.1 | hsa-miR-205-5p | CENPF |
| AC067959.1 | hsa-miR-205-5p | ESRRG |
| AC092667.2 | hsa-miR-204-5p | HMGA2 |
| AC092667.2 | hsa-miR-204-5p | HOXC8 |
| AC108676.1 | hsa-miR-143-3p | COL1A1 |
| AC108676.1 | hsa-miR-143-3p | SERPINE1 |
| AL133493.2 | hsa-miR-143-3p | COL1A1 |
| AL133493.2 | hsa-miR-5589-5p | GRIK3 |
| AL133493.2 | hsa-miR-143-3p | SERPINE1 |
| AP000695.6 | hsa-miR-5683 | EPGN |
| ARHGEF26-AS1 | hsa-miR-204-5p | HMGA2 |
| ARHGEF26-AS1 | hsa-miR-204-5p | HOXC8 |
| BVES-AS1 | hsa-miR-145-5p | MEST |
| BVES-AS1 | hsa-miR-145-5p | SERPINE1 |
| CCDC144NL-AS1 | hsa-miR-1-3p | ADAM12 |
| CCDC144NL-AS1 | hsa-miR-3662 | ADAMTS18 |
| CCDC144NL-AS1 | hsa-miR-372-3p | ATAD2 |
| CCDC144NL-AS1 | hsa-miR-205-5p | BAMBI |
| CCDC144NL-AS1 | hsa-miR-512-3p | CADM2 |
| CCDC144NL-AS1 | hsa-miR-205-5p | CENPF |
| CCDC144NL-AS1 | hsa-miR-143-3p | COL1A1 |
| CCDC144NL-AS1 | hsa-miR-205-5p | ESRRG |
| CCDC144NL-AS1 | hsa-miR-1-3p | HAND2 |
| CCDC144NL-AS1 | hsa-miR-3662 | HMGA2 |
| CCDC144NL-AS1 | hsa-miR-512-3p | KCNB1 |
| CCDC144NL-AS1 | hsa-miR-145-5p | MEST |
| CCDC144NL-AS1 | hsa-miR-1-3p | MET |
| CCDC144NL-AS1 | hsa-miR-143-3p | SERPINE1 |
| CCDC144NL-AS1 | hsa-miR-145-5p | SERPINE1 |
| CCDC144NL-AS1 | hsa-miR-1304-3p | SHISA9 |
| CCDC144NL-AS1 | hsa-miR-3662 | SHISA9 |
| CCDC144NL-AS1 | hsa-miR-3662 | SYNM |
| CCDC144NL-AS1 | hsa-miR-1304-3p | SYNPO2L |
| CTD-2314B22.1 | hsa-miR-204-5p | HMGA2 |
| CTD-2314B22.1 | hsa-miR-204-5p | HOXC8 |
| CTD-2651B20.6 | hsa-miR-204-5p | HMGA2 |
| CTD-2651B20.6 | hsa-miR-204-5p | HOXC8 |
| DLGAP1-AS2 | hsa-miR-143-3p | COL1A1 |
| DLGAP1-AS2 | hsa-miR-143-3p | SERPINE1 |
| DLX6-AS1 | hsa-miR-1-3p | ADAM12 |
| DLX6-AS1 | hsa-miR-3662 | ADAMTS18 |
| DLX6-AS1 | hsa-miR-143-3p | COL1A1 |
| DLX6-AS1 | hsa-miR-5683 | EPGN |
| DLX6-AS1 | hsa-miR-1-3p | HAND2 |
| DLX6-AS1 | hsa-miR-204-5p | HMGA2 |
| DLX6-AS1 | hsa-miR-3662 | HMGA2 |
| DLX6-AS1 | hsa-miR-204-5p | HOXC8 |
| DLX6-AS1 | hsa-miR-145-5p | MEST |
| DLX6-AS1 | hsa-miR-1-3p | MET |
| DLX6-AS1 | hsa-miR-6507-5p | PLA2G4D |
| DLX6-AS1 | hsa-miR-143-3p | SERPINE1 |
| DLX6-AS1 | hsa-miR-145-5p | SERPINE1 |
| DLX6-AS1 | hsa-miR-3662 | SHISA9 |
| DLX6-AS1 | hsa-miR-944 | STRIP2 |
| DLX6-AS1 | hsa-miR-3662 | SYNM |
| DLX6-AS1 | hsa-miR-6507-5p | ZIC5 |
| FOXD2-AS1 | hsa-miR-1-3p | ADAM12 |
| FOXD2-AS1 | hsa-miR-1-3p | HAND2 |
| FOXD2-AS1 | hsa-miR-1-3p | MET |
| GS1-600G8.5 | hsa-miR-3662 | ADAMTS18 |
| GS1-600G8.5 | hsa-miR-3662 | HMGA2 |
| GS1-600G8.5 | hsa-miR-3662 | SHISA9 |
| GS1-600G8.5 | hsa-miR-3662 | SYNM |
| HNF4A-AS1 | hsa-miR-143-3p | COL1A1 |
| HNF4A-AS1 | hsa-miR-143-3p | SERPINE1 |
| HOTAIR | hsa-miR-204-5p | HMGA2 |
| HOTAIR | hsa-miR-204-5p | HOXC8 |
| HOXA10-AS | hsa-miR-205-5p | BAMBI |
| HOXA10-AS | hsa-miR-205-5p | CENPF |
| HOXA10-AS | hsa-miR-205-5p | ESRRG |
| HULC | hsa-miR-372-3p | ATAD2 |
| IL12A-AS1 | hsa-miR-1-3p | ADAM12 |
| IL12A-AS1 | hsa-miR-1-3p | HAND2 |
| IL12A-AS1 | hsa-miR-1-3p | MET |
| L29074.3 | hsa-miR-1304-3p | SHISA9 |
| L29074.3 | hsa-miR-1304-3p | SYNPO2L |
| LINC00682 | hsa-miR-944 | STRIP2 |
| LINC00941 | hsa-miR-205-5p | BAMBI |
| LINC00941 | hsa-miR-205-5p | CENPF |
| LINC00941 | hsa-miR-205-5p | ESRRG |
| LINC00941 | hsa-miR-944 | STRIP2 |
| LINC00982 | hsa-miR-3662 | ADAMTS18 |
| LINC00982 | hsa-miR-4728-3p | FOXE1 |
| LINC00982 | hsa-miR-3662 | HMGA2 |
| LINC00982 | hsa-miR-25-5p | LY6H |
| LINC00982 | hsa-miR-3662 | SHISA9 |
| LINC00982 | hsa-miR-3662 | SYNM |
| LINC01021 | hsa-miR-1-3p | ADAM12 |
| LINC01021 | hsa-miR-1-3p | HAND2 |
| LINC01021 | hsa-miR-1-3p | MET |
| LINC01021 | hsa-miR-5589-3p | SLC5A7 |
| LINC01146 | hsa-miR-143-3p | COL1A1 |
| LINC01146 | hsa-miR-143-3p | SERPINE1 |
| LINC01194 | hsa-miR-3662 | ADAMTS18 |
| LINC01194 | hsa-miR-512-3p | CADM2 |
| LINC01194 | hsa-miR-3662 | HMGA2 |
| LINC01194 | hsa-miR-512-3p | KCNB1 |
| LINC01194 | hsa-miR-3662 | SHISA9 |
| LINC01194 | hsa-miR-5589-3p | SLC5A7 |
| LINC01194 | hsa-miR-3662 | SYNM |
| LINC01234 | hsa-miR-5589-5p | GRIK3 |
| LINC01234 | hsa-miR-5589-3p | SLC5A7 |
| LINC01235 | hsa-miR-3662 | ADAMTS18 |
| LINC01235 | hsa-miR-3662 | HMGA2 |
| LINC01235 | hsa-miR-3662 | SHISA9 |
| LINC01235 | hsa-miR-944 | STRIP2 |
| LINC01235 | hsa-miR-3662 | SYNM |
| LINC01287 | hsa-miR-196a-5p | EPHA7 |
| LINC01287 | hsa-miR-5589-5p | GRIK3 |
| LINC01287 | hsa-miR-196a-5p | HMGA2 |
| LINC01287 | hsa-miR-204-5p | HMGA2 |
| LINC01287 | hsa-miR-196a-5p | HOXA9 |
| LINC01287 | hsa-miR-196a-5p | HOXC8 |
| LINC01287 | hsa-miR-204-5p | HOXC8 |
| LINC01287 | hsa-miR-196a-5p | IGF2BP1 |
| LINC01287 | hsa-miR-1269a | KCNMB1 |
| LINC01287 | hsa-miR-1269a | KCNMB1 |
| LINC01287 | hsa-miR-145-5p | MEST |
| LINC01287 | hsa-miR-196a-5p | NRXN1 |
| LINC01287 | hsa-miR-145-5p | SERPINE1 |
| LINC01518 | hsa-miR-1-3p | ADAM12 |
| LINC01518 | hsa-miR-205-5p | BAMBI |
| LINC01518 | hsa-miR-205-5p | CENPF |
| LINC01518 | hsa-miR-205-5p | ESRRG |
| LINC01518 | hsa-miR-1-3p | HAND2 |
| LINC01518 | hsa-miR-1-3p | MET |
| NPSR1-AS1 | hsa-miR-5683 | EPGN |
| NPSR1-AS1 | hsa-miR-1304-3p | SHISA9 |
| NPSR1-AS1 | hsa-miR-5589-3p | SLC5A7 |
| NPSR1-AS1 | hsa-miR-1304-3p | SYNPO2L |
| PART1 | hsa-miR-1-3p | ADAM12 |
| PART1 | hsa-miR-3662 | ADAMTS18 |
| PART1 | hsa-miR-7974 | ALPI |
| PART1 | hsa-miR-143-3p | COL1A1 |
| PART1 | hsa-miR-1-3p | HAND2 |
| PART1 | hsa-miR-3662 | HMGA2 |
| PART1 | hsa-miR-1-3p | MET |
| PART1 | hsa-miR-143-3p | SERPINE1 |
| PART1 | hsa-miR-3662 | SHISA9 |
| PART1 | hsa-miR-3662 | SYNM |
| PP14571 | hsa-miR-5589-5p | GRIK3 |
| PWAR6 | hsa-miR-1-3p | ADAM12 |
| PWAR6 | hsa-miR-3662 | ADAMTS18 |
| PWAR6 | hsa-miR-372-3p | ATAD2 |
| PWAR6 | hsa-miR-512-3p | CADM2 |
| PWAR6 | hsa-miR-143-3p | COL1A1 |
| PWAR6 | hsa-miR-4728-3p | FOXE1 |
| PWAR6 | hsa-miR-1-3p | HAND2 |
| PWAR6 | hsa-miR-3662 | HMGA2 |
| PWAR6 | hsa-miR-512-3p | KCNB1 |
| PWAR6 | hsa-miR-145-5p | MEST |
| PWAR6 | hsa-miR-1-3p | MET |
| PWAR6 | hsa-miR-6507-5p | PLA2G4D |
| PWAR6 | hsa-miR-143-3p | SERPINE1 |
| PWAR6 | hsa-miR-145-5p | SERPINE1 |
| PWAR6 | hsa-miR-3662 | SHISA9 |
| PWAR6 | hsa-miR-3662 | SYNM |
| PWAR6 | hsa-miR-6507-5p | ZIC5 |
| RBMS3-AS3 | hsa-miR-204-5p | HMGA2 |
| RBMS3-AS3 | hsa-miR-204-5p | HOXC8 |
| RNF144A-AS1 | hsa-miR-205-5p | BAMBI |
| RNF144A-AS1 | hsa-miR-512-3p | CADM2 |
| RNF144A-AS1 | hsa-miR-205-5p | CENPF |
| RNF144A-AS1 | hsa-miR-205-5p | ESRRG |
| RNF144A-AS1 | hsa-miR-512-3p | KCNB1 |
| RP1-102K2.8 | hsa-miR-1304-3p | SHISA9 |
| RP1-102K2.8 | hsa-miR-1304-3p | SYNPO2L |
| RP11-1103G16.1 | hsa-miR-1-3p | ADAM12 |
| RP11-1103G16.1 | hsa-miR-1-3p | HAND2 |
| RP11-1103G16.1 | hsa-miR-1-3p | MET |
| RP11-1143G9.5 | hsa-miR-3662 | ADAMTS18 |
| RP11-1143G9.5 | hsa-miR-204-5p | HMGA2 |
| RP11-1143G9.5 | hsa-miR-3662 | HMGA2 |
| RP11-1143G9.5 | hsa-miR-204-5p | HOXC8 |
| RP11-1143G9.5 | hsa-miR-6507-5p | PLA2G4D |
| RP11-1143G9.5 | hsa-miR-3662 | SHISA9 |
| RP11-1143G9.5 | hsa-miR-5589-3p | SLC5A7 |
| RP11-1143G9.5 | hsa-miR-3662 | SYNM |
| RP11-1143G9.5 | hsa-miR-6507-5p | ZIC5 |
| RP11-134N1.2 | hsa-miR-143-3p | COL1A1 |
| RP11-134N1.2 | hsa-miR-143-3p | SERPINE1 |
| RP11-145A3.1 | hsa-miR-3662 | ADAMTS18 |
| RP11-145A3.1 | hsa-miR-3662 | HMGA2 |
| RP11-145A3.1 | hsa-miR-3662 | SHISA9 |
| RP11-145A3.1 | hsa-miR-3662 | SYNM |
| RP11-161I6.2 | hsa-miR-767-5p | COL10A1 |
| RP11-161I6.2 | hsa-miR-767-5p | COL3A1 |
| RP11-161I6.2 | hsa-miR-4728-3p | FOXE1 |
| RP11-197K6.1 | hsa-miR-1269a | KCNMB1 |
| RP11-197K6.1 | hsa-miR-1269a | KCNMB1 |
| RP11-197K6.1 | hsa-miR-675-3p | OPCML |
| RP11-197K6.1 | hsa-miR-675-3p | PLP1 |
| RP11-332J15.4 | hsa-miR-204-5p | HMGA2 |
| RP11-332J15.4 | hsa-miR-204-5p | HOXC8 |
| RP11-354K4.2 | hsa-miR-944 | STRIP2 |
| RP11-370A5.2 | hsa-miR-143-3p | COL1A1 |
| RP11-370A5.2 | hsa-miR-143-3p | SERPINE1 |
| RP11-428O18.6 | hsa-miR-5589-3p | SLC5A7 |
| RP11-492E3.2 | hsa-miR-5589-5p | GRIK3 |
| RP11-532F6.3 | hsa-miR-3662 | ADAMTS18 |
| RP11-532F6.3 | hsa-miR-3662 | HMGA2 |
| RP11-532F6.3 | hsa-miR-3662 | SHISA9 |
| RP11-532F6.3 | hsa-miR-3662 | SYNM |
| RP11-575F12.3 | hsa-miR-1-3p | ADAM12 |
| RP11-575F12.3 | hsa-miR-1-3p | HAND2 |
| RP11-575F12.3 | hsa-miR-1-3p | MET |
| RP11-576I22.2 | hsa-miR-1-3p | ADAM12 |
| RP11-576I22.2 | hsa-miR-205-5p | BAMBI |
| RP11-576I22.2 | hsa-miR-205-5p | CENPF |
| RP11-576I22.2 | hsa-miR-143-3p | COL1A1 |
| RP11-576I22.2 | hsa-miR-5683 | EPGN |
| RP11-576I22.2 | hsa-miR-205-5p | ESRRG |
| RP11-576I22.2 | hsa-miR-1-3p | HAND2 |
| RP11-576I22.2 | hsa-miR-1-3p | MET |
| RP11-576I22.2 | hsa-miR-143-3p | SERPINE1 |
| RP11-576I22.2 | hsa-miR-944 | STRIP2 |
| RP11-758M4.4 | hsa-miR-204-5p | HMGA2 |
| RP11-758M4.4 | hsa-miR-204-5p | HOXC8 |
| RP11-80H5.2 | hsa-miR-205-5p | BAMBI |
| RP11-80H5.2 | hsa-miR-205-5p | CENPF |
| RP11-80H5.2 | hsa-miR-205-5p | ESRRG |
| RP11-874J12.4 | hsa-miR-196a-5p | EPHA7 |
| RP11-874J12.4 | hsa-miR-196a-5p | HMGA2 |
| RP11-874J12.4 | hsa-miR-196a-5p | HOXA9 |
| RP11-874J12.4 | hsa-miR-196a-5p | HOXC8 |
| RP11-874J12.4 | hsa-miR-196a-5p | IGF2BP1 |
| RP11-874J12.4 | hsa-miR-196a-5p | NRXN1 |
| RP11-96C23.14 | hsa-miR-205-5p | BAMBI |
| RP11-96C23.14 | hsa-miR-205-5p | CENPF |
| RP11-96C23.14 | hsa-miR-205-5p | ESRRG |
| RP1-60O19.1 | hsa-miR-4728-3p | FOXE1 |
| RP1-60O19.1 | hsa-miR-5589-5p | GRIK3 |
| RP1-60O19.1 | hsa-miR-145-5p | MEST |
| RP1-60O19.1 | hsa-miR-145-5p | SERPINE1 |
| RP1-90G24.6 | hsa-miR-944 | STRIP2 |
| RP5-1120P11.1 | hsa-miR-145-5p | MEST |
| RP5-1120P11.1 | hsa-miR-145-5p | SERPINE1 |
| RP5-884M6.1 | hsa-miR-204-5p | HMGA2 |
| RP5-884M6.1 | hsa-miR-204-5p | HOXC8 |
| RP6-91H8.3 | hsa-miR-5589-5p | GRIK3 |
| SOX21-AS1 | hsa-miR-3662 | ADAMTS18 |
| SOX21-AS1 | hsa-miR-205-5p | BAMBI |
| SOX21-AS1 | hsa-miR-205-5p | CENPF |
| SOX21-AS1 | hsa-miR-205-5p | ESRRG |
| SOX21-AS1 | hsa-miR-3662 | HMGA2 |
| SOX21-AS1 | hsa-miR-3662 | SHISA9 |
| SOX21-AS1 | hsa-miR-3662 | SYNM |
| ST8SIA6-AS1 | hsa-miR-145-5p | MEST |
| ST8SIA6-AS1 | hsa-miR-145-5p | SERPINE1 |
| TINCR | hsa-miR-5589-5p | GRIK3 |
| TINCR | hsa-miR-1304-3p | SHISA9 |
| TINCR | hsa-miR-1304-3p | SYNPO2L |
| TMEM220-AS1 | hsa-miR-767-5p | COL10A1 |
| TMEM220-AS1 | hsa-miR-767-5p | COL3A1 |
| TSPEAR-AS2 | hsa-miR-5589-3p | SLC5A7 |
| UCA1 | hsa-miR-1-3p | ADAM12 |
| UCA1 | hsa-miR-7974 | ALPI |
| UCA1 | hsa-miR-143-3p | COL1A1 |
| UCA1 | hsa-miR-5589-5p | GRIK3 |
| UCA1 | hsa-miR-1-3p | HAND2 |
| UCA1 | hsa-miR-1-3p | MET |
| UCA1 | hsa-miR-143-3p | SERPINE1 |
| UG0898H09 | hsa-miR-3662 | ADAMTS18 |
| UG0898H09 | hsa-miR-3662 | HMGA2 |
| UG0898H09 | hsa-miR-6507-5p | PLA2G4D |
| UG0898H09 | hsa-miR-3662 | SHISA9 |
| UG0898H09 | hsa-miR-944 | STRIP2 |
| UG0898H09 | hsa-miR-3662 | SYNM |
| UG0898H09 | hsa-miR-6507-5p | ZIC5 |
| XX-C2158C6.3 | hsa-miR-204-5p | HMGA2 |
| XX-C2158C6.3 | hsa-miR-204-5p | HOXC8 |

**Table S2. The expression levels of CCDC144NL-AS1, hsa-miR-145-5p and SERPINE1 in the patients for correlation analysis.**

| Patient ID | CCDC144NL-AS1 [Log_2_(TPM+1)] | hsa-miR-145-5p [Log_2_(TPM+1)] | SERPINE1 [Log_2_(TPM+1)] |
| --- | --- | --- | --- |
| TCGA-B7-5818-01A-11R-1602-13 | 4.352292 | 0.069581 | 11.46879 |
| TCGA-BR-4253-01A-01R-1131-13 | 4.34958 | 0.074875 | 13.59172 |
| TCGA-BR-4267-01A-01R-1131-13 | 4.628397 | 0.077555 | 11.37595 |
| TCGA-BR-4279-01A-01R-1131-13 | 6.315868 | 0.54479 | 12.46603 |
| TCGA-BR-6452-01A-12R-1802-13 | 4.355359 | 0.161063 | 13.58326 |
| TCGA-BR-6453-01A-11R-1802-13 | 4.873569 | 0.208583 | 10.54464 |
| TCGA-BR-6457-01A-21R-1802-13 | 5.452401 | 0.31668 | 9.835654 |
| TCGA-BR-6458-01A-11R-1802-13 | 6.044932 | 0.308944 | 9.526334 |
| TCGA-BR-6563-01A-13R-2055-13 | 4.113435 | 0.680913 | 11.83822 |
| TCGA-BR-6564-01A-12R-1884-13 | 3.972882 | 0.890135 | 12.39339 |
| TCGA-BR-6565-01A-11R-1802-13 | 4.126647 | 0.168268 | 11.43011 |
| TCGA-BR-6566-01A-11R-1802-13 | 3.726909 | 0.153395 | 10.74418 |
| TCGA-BR-6705-01A-12R-1884-13 | 6.329382 | 0.521985 | 10.42213 |
| TCGA-BR-6707-01A-11R-1884-13 | 3.187924 | 0.148631 | 13.69403 |
| TCGA-BR-6710-01A-11R-1884-13 | 4.17324 | 0.185265 | 12.90539 |
| TCGA-BR-6802-01A-11R-1884-13 | 5.044827 | 0.074908 | 11.36472 |
| TCGA-BR-6803-01A-11R-1884-13 | 3.337855 | 0.391007 | 11.55772 |
| TCGA-BR-6852-01A-11R-1884-13 | 5.468758 | 0.130865 | 12.41854 |
| TCGA-BR-7707-01A-11R-2055-13 | 4.546663 | 0.042437 | 12.33079 |
| TCGA-BR-7715-01A-11R-2055-13 | 4.431466 | 0.33896 | 10.76767 |
| TCGA-BR-7716-01A-21R-2055-13 | 4.434029 | 0.560869 | 13.39904 |
| TCGA-BR-7722-01A-31R-2203-13 | 3.874012 | 0.273621 | 12.89839 |
| TCGA-BR-7851-01A-11R-2203-13 | 4.750304 | 0.112488 | 13.19998 |
| TCGA-BR-7901-01A-11R-2203-13 | 6.292448 | 0.540537 | 11.13203 |
| TCGA-BR-7957-01A-11R-2203-13 | 5.569671 | 0.381715 | 13.03364 |
| TCGA-BR-8059-01A-11R-2343-13 | 5.296351 | 0.24924 | 11.47392 |
| TCGA-BR-8060-01A-11R-2343-13 | 6.568883 | 0.164924 | 11.00742 |
| TCGA-BR-8080-01A-11R-2343-13 | 5.644506 | 0.34863 | 12.47587 |
| TCGA-BR-8286-01A-12R-2343-13 | 3.425452 | 0.246208 | 10.95128 |
| TCGA-BR-8289-01A-11R-2343-13 | 3.79138 | 0.282419 | 10.51979 |
| TCGA-BR-8291-01A-11R-2343-13 | 6.699155 | 0.303771 | 11.12125 |
| TCGA-BR-8296-01A-11R-2343-13 | 5.610322 | 0.418305 | 9.174253 |
| TCGA-BR-8297-01A-12R-2343-13 | 4.092134 | 0.211514 | 11.89389 |
| TCGA-BR-8361-01A-11R-2343-13 | 4.521511 | 0.306109 | 12.41427 |
| TCGA-BR-8364-01A-11R-2343-13 | 7.112419 | 0.679025 | 11.53992 |
| TCGA-BR-8365-01A-21R-2343-13 | 5.154545 | 0.56982 | 11.45926 |
| TCGA-BR-8367-01A-11R-2343-13 | 4.053962 | 0.268651 | 12.16635 |
| TCGA-BR-8368-01A-11R-2343-13 | 3.599846 | 0.096479 | 10.93219 |
| TCGA-BR-8369-01A-11R-2343-13 | 5.108428 | 0.179035 | 12.5941 |
| TCGA-BR-8371-01A-11R-2343-13 | 2.443534 | 0.155447 | 11.43109 |
| TCGA-BR-8373-01A-11R-2343-13 | 4.833753 | 0.4296 | 12.34992 |
| TCGA-BR-8381-01A-11R-2402-13 | 4.973766 | 0.492534 | 10.63843 |
| TCGA-BR-8382-01A-11R-2402-13 | 3.668597 | 0.309707 | 12.10124 |
| TCGA-BR-8384-01A-21R-2402-13 | 5.16845 | 0.221382 | 10.265 |
| TCGA-BR-8483-01A-31R-2402-13 | 2.896509 | 0.269523 | 12.26033 |
| TCGA-BR-8484-01A-11R-2402-13 | 4.430445 | 0.205921 | 10.59293 |
| TCGA-BR-8485-01A-11R-2402-13 | 5.577281 | 1.159765 | 10.61942 |
| TCGA-BR-8487-01A-11R-2402-13 | 3.744014 | 0.194044 | 11.46171 |
| TCGA-BR-8588-01A-11R-2402-13 | 5.717424 | 0.195461 | 12.82997 |
| TCGA-BR-8590-01A-11R-2402-13 | 4.688389 | 0.623222 | 12.91012 |
| TCGA-BR-8591-01A-11R-2402-13 | 4.950361 | 0.610799 | 12.52471 |
| TCGA-BR-8592-01A-11R-2402-13 | 4.55964 | 0.456 | 11.28022 |
| TCGA-BR-8676-01A-11R-2402-13 | 3.770792 | 0.061579 | 12.4005 |
| TCGA-BR-8677-01A-11R-2402-13 | 6.370822 | 0.327706 | 9.166386 |
| TCGA-BR-8680-01A-11R-2402-13 | 2.306238 | 0.086066 | 12.42065 |
| TCGA-BR-8682-01A-11R-2402-13 | 2.844449 | 0.192917 | 11.18851 |
| TCGA-BR-8683-01A-11R-2402-13 | 5.366819 | 0.340397 | 11.55324 |
| TCGA-BR-8686-01A-11R-2402-13 | 5.359725 | 0.066058 | 12.87414 |
| TCGA-BR-8687-01A-11R-2402-13 | 4.371146 | 0.336767 | 10.29361 |
| TCGA-BR-8690-01A-11R-2402-13 | 3.588825 | 0.499363 | 10.46149 |
| TCGA-CD-5798-01A-11R-1602-13 | 5.062462 | 0.454209 | 12.40197 |
| TCGA-CD-5799-01A-11R-1602-13 | 5.987295 | 0.148293 | 11.40833 |
| TCGA-CD-5803-01A-11R-1602-13 | 6.429019 | 0.445666 | 10.71684 |
| TCGA-CD-8524-01A-11R-2343-13 | 5.517114 | 0.739973 | 12.04735 |
| TCGA-CD-8528-01A-11R-2343-13 | 3.035191 | 0.075981 | 12.8242 |
| TCGA-CD-8529-01A-11R-2343-13 | 5.923833 | 0.280054 | 11.58584 |
| TCGA-CD-8530-01A-11R-2343-13 | 6.362933 | 0.640257 | 11.84527 |
| TCGA-CD-8531-01A-11R-2343-13 | 4.305964 | 0.443098 | 9.758077 |
| TCGA-CD-8532-01A-11R-2343-13 | 4.596888 | 0.243771 | 12.01477 |
| TCGA-CD-8533-01A-11R-2343-13 | 3.181559 | 0.745082 | 10.52662 |
| TCGA-CD-8534-01A-11R-2343-13 | 1.738677 | 0.151426 | 11.94848 |
| TCGA-CD-8535-01A-11R-2343-13 | 4.15454 | 0.08516 | 12.00176 |
| TCGA-CG-4301-01A-01R-1157-13 | 4.990046 | 0.513607 | 12.96048 |
| TCGA-CG-4305-01A-01R-1157-13 | 5.230764 | 0.452486 | 11.45889 |
| TCGA-CG-4438-01A-01R-1157-13 | 3.81004 | 0.174677 | 13.09487 |
| TCGA-CG-4441-01A-01R-1802-13 | 4.458573 | 0.295186 | 12.90031 |
| TCGA-CG-4443-01A-01R-1157-13 | 2.399426 | 0.083578 | 13.67726 |
| TCGA-CG-4444-01A-01R-1157-13 | 4.031192 | 0.21345 | 11.2423 |
| TCGA-CG-4465-01A-01R-1157-13 | 3.442367 | 0.126629 | 10.62997 |
| TCGA-CG-4466-01A-01R-1157-13 | 4.341036 | 0.131042 | 11.13927 |
| TCGA-CG-4475-01A-01R-1157-13 | 6.737038 | 0.339116 | 10.87556 |
| TCGA-CG-4477-01A-01R-1157-13 | 5.726825 | 0.233916 | 10.51315 |
| TCGA-CG-5717-01A-11R-1602-13 | 4.300744 | 0.118927 | 12.16503 |
| TCGA-CG-5720-01A-11R-1602-13 | 5.960817 | 0.123872 | 11.31879 |
| TCGA-CG-5721-01A-11R-1602-13 | 4.108172 | 1.133501 | 10.12539 |
| TCGA-CG-5722-01A-21R-1602-13 | 4.789005 | 0.147865 | 11.50678 |
| TCGA-CG-5724-01A-11R-1602-13 | 5.445783 | 0.111611 | 11.87756 |
| TCGA-CG-5725-01A-11R-1602-13 | 4.336201 | 1.541082 | 10.80867 |
| TCGA-CG-5726-01A-11R-1602-13 | 2.404723 | 0.219947 | 11.68374 |
| TCGA-CG-5732-01A-11R-1602-13 | 2.657639 | 0.09673 | 10.99915 |
| TCGA-D7-5577-01A-01R-1602-13 | 5.441701 | 0.135507 | 11.94033 |
| TCGA-D7-5578-01A-01R-1602-13 | 3.6125 | 0.198309 | 12.54806 |
| TCGA-D7-6520-01A-11R-1802-13 | 3.624913 | 0.494713 | 11.1609 |
| TCGA-D7-6522-01A-11R-1802-13 | 4.572535 | 0.389734 | 11.86806 |
| TCGA-D7-6524-01A-11R-1802-13 | 5.946829 | 0.424077 | 10.16147 |
| TCGA-D7-6525-01A-11R-1802-13 | 7.743637 | 0.168453 | 10.59094 |
| TCGA-D7-6526-01A-11R-1802-13 | 4.662408 | 0.700783 | 11.81608 |
| TCGA-D7-6527-01A-11R-1802-13 | 2.770337 | 0.11771 | 11.10305 |
| TCGA-D7-6528-01A-11R-1802-13 | 4.593617 | 0.099278 | 11.3081 |
| TCGA-D7-6815-01A-11R-1884-13 | 3.01568 | 0.203803 | 11.07744 |
| TCGA-D7-6818-01A-11R-1884-13 | 5.063071 | 0.154536 | 10.80376 |
| TCGA-D7-8570-01A-11R-2343-13 | 4.736119 | 0.217328 | 13.16972 |
| TCGA-D7-8573-01A-11R-2343-13 | 2.212185 | 0.127847 | 12.31367 |
| TCGA-D7-8574-01A-13R-2343-13 | 3.406348 | 0.365354 | 13.23036 |
| TCGA-D7-8575-01A-11R-2343-13 | 4.764592 | 0.182895 | 13.05548 |
| TCGA-D7-8576-01A-11R-2343-13 | 3.48982 | 0.157505 | 11.16505 |
| TCGA-D7-8579-01A-11R-2343-13 | 3.510123 | 0.180626 | 11.97672 |
| TCGA-FP-7735-01A-11R-2055-13 | 5.480585 | 0.066562 | 12.1076 |
| TCGA-FP-7829-01A-11R-2055-13 | 3.134874 | 0.152768 | 11.43439 |
| TCGA-FP-7916-01A-11R-2203-13 | 2.96601 | 0.371062 | 13.91413 |
| TCGA-FP-7998-01A-11R-2203-13 | 3.350151 | 0.15075 | 11.45868 |
| TCGA-FP-8099-01A-11R-2343-13 | 3.263557 | 0.2191 | 12.59611 |
| TCGA-FP-8209-01A-11R-2343-13 | 5.050713 | 0.285091 | 12.41587 |
| TCGA-FP-8210-01A-11R-2343-13 | 3.894748 | 0.54529 | 12.04765 |
| TCGA-FP-8211-01A-11R-2343-13 | 3.122404 | 0.115543 | 10.69865 |
| TCGA-HU-8244-01A-11R-2343-13 | 1.202224 | 0.013307 | 12.02514 |
| TCGA-HU-8604-01A-11R-2402-13 | 6.555167 | 0.295606 | 14.0178 |
| TCGA-HU-8608-01A-11R-2402-13 | 4.362384 | 0.036166 | 12.28409 |
| TCGA-IN-7806-01A-11R-2055-13 | 2.600902 | 0.183856 | 14.00039 |
| TCGA-IN-8462-01A-11R-2343-13 | 5.629307 | 0.180628 | 12.38351 |
| TCGA-IP-7968-01A-11R-2203-13 | 5.818875 | 0.154452 | 11.23187 |

**Table S3. Univariate analysis of overall survival in STAD patients stratified based on clinical characteristics.**

| Factor | Variable | N | CCDC144NL-AS1 Expression (Median) | *P* value | SERPINE1 Expression (Median) | *P* value | Overall survival | | |
| --- | --- | --- | --- | --- | --- | --- | --- | --- | --- |
|  |  |  |  |  |  |  | Months (Median) | 95% CI (Median) | P value |
| Age | ＞65 | 207 | 0.511 | 0.289 | 5.883 | 0.425 | 22.500 | 16.906-28.094 | **0.002** |
|  | ≤65 | 164 | 0.456 |  | 5.728 |  | 46.900 | 23.492-70.308 |  |
| Gender | Male | 241 | 0.462 | 0.191 | 5.760 | 0.704 | 28.967 | 14.317-43.616 | 0.195 |
|  | Female | 134 | 0.524 |  | 5.871 |  | 34.767 | 23.562-45.972 |  |
| Pathologic stage | Stage I-II | 164 | 0.457 | 0.784 | 5.724 | 0.153 | 60.367 | 33.031-87.702 | **0.001** |
|  | Stage III-IV | 168 | 0.480 |  | 5.898 |  | 23.733 | 17.629-29.839 |  |
| Histologic stage | G1-G2 | 147 | 0.445 | 0.064 | 5.694 | **0.013** | 43.133 | 16.863-69.404 | 0.109 |
|  | G3 | 219 | 0.496 |  | 5.901 |  | 26.700 | 15.999-37.401 |  |
| T stage | T1-T2 | 99 | 0.364 | **0.011** | 5.656 | 0.124 | 70.000 | 22.351-117.649 | **0.008** |
|  | T3-T4 | 268 | 0.500 |  | 5.889 |  | 26.400 | 17.424-35.376 |  |
| N stage | N0 | 111 | 0.451 | 0.868 | 5.733 | 0.361 | 60.367 | 27.784-92.949 | **0.005** |
|  | N1-N3 | 246 | 0.468 |  | 5.817 |  | 25.533 | 19.854-31.212 |  |
| M stage | M0 | 330 | 0.466 | 0.907 | 5.761 | 0.710 | 34.767 | 22.220-47.313 | **0.003** |
|  | M1 | 25 | 0.504 |  | 5.982 |  | 12.200 | 6.768-17.632 |  |

**Table S4. Correlation analysis between SERPINE1 and biomarkers of immune cells.**

| Cell | STAD | |
| --- | --- | --- |
|  | Correlation coefficient (Pearson) | *p* value (Pearson) |
| aDC | 0.156 | **0.002** |
| B cells | 0.018 | 0.722 |
| CD8 T cells | 0.217 | **<0.001** |
| Cytotoxic cells | 0.214 | **<0.001** |
| DC | 0.337 | **<0.001** |
| Eosinophils | 0.214 | **<0.001** |
| iDC | 0.281 | **<0.001** |
| Macrophages | 0.448 | **<0.001** |
| Mast cells | 0.251 | **<0.001** |
| Neutrophils | 0.389 | **<0.001** |
| NK CD56bright cells | 0.025 | 0.626 |
| NK CD56dim cells | 0.236 | **<0.001** |
| NK cells | 0.305 | **<0.001** |
| pDC | 0.233 | **<0.001** |
| T cells | 0.106 | **0.040** |
| T helper cells | -0.012 | 0.819 |
| Tcm | 0.059 | 0.254 |
| Tem | 0.343 | **<0.001** |
| Tfh | 0.169 | **0.001** |
| Tgd | 0.101 | 0.051 |
| Th1 cells | 0.461 | **<0.001** |
| Th17 cells | -0.122 | **0.018** |
| Th2 cells | 0.026 | 0.619 |
| Treg | 0.155 | **0.003** |


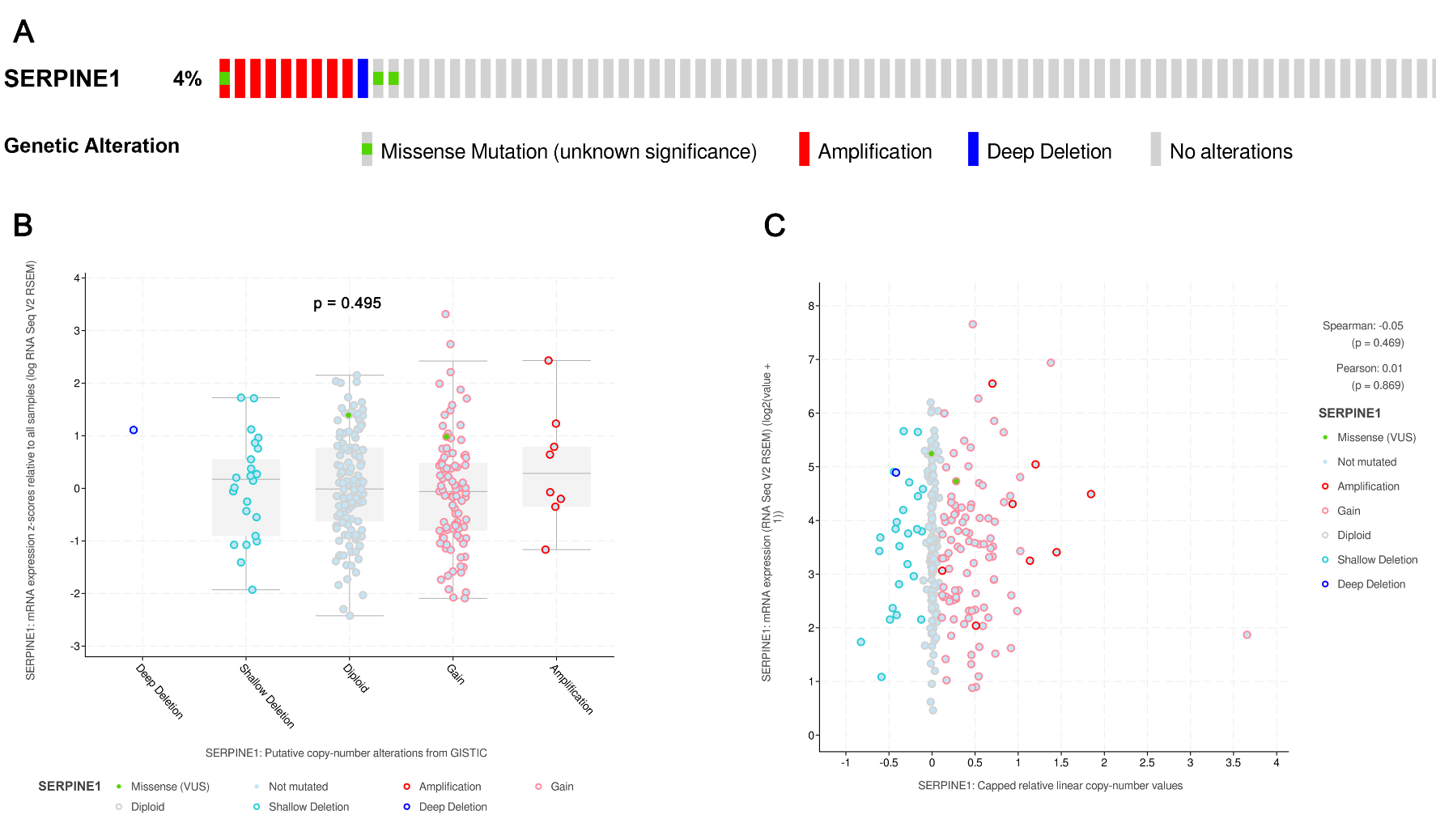


**Figure S1** The mutation status of SERPINE1 in STAD. (A) The distribution of SERPINE1 genomic alterations in the TCGA-STAD dataset is shown on the cBioPortal OncoPrint plot. (B) The correlation analysis between SERPINE1 copy number and mRNA expression showed in box-point plot. (C) The correlation analysis between SERPINE1 copy number and mRNA expression showed in correlation plot.
